# Transcriptome Profiling-Based Analysis of Carbohydrate-Active Enzymes in *Aspergillus terreus* Involved in Plant Biomass Degradation

**DOI:** 10.3389/fbioe.2020.564527

**Published:** 2020-10-06

**Authors:** Camila L. Corrêa, Glaucia E. O. Midorikawa, Edivaldo Ximenes Ferreira Filho, Eliane Ferreira Noronha, Gabriel S. C. Alves, Roberto Coiti Togawa, Orzenil Bonfim Silva-Junior, Marcos Mota do Carmo Costa, Priscila Grynberg, Robert N. G. Miller

**Affiliations:** ^1^Departamento de Biologia Celular, Universidade de Brasília, Campus Universitário Darcy Ribeiro, Brasília, Brazil; ^2^Embrapa Recursos Genéticos e Biotecnologia, Parque Estação Biológica – PqEB, Brasília, Brazil

**Keywords:** *Aspergillus terreus*, lignocellulosic biomass, biorefinery, transcriptome, carbohydrate-active enzymes

## Abstract

Given the global abundance of plant biomass residues, potential exists in biorefinery-based applications with lignocellulolytic fungi. Frequently isolated from agricultural cellulosic materials, *Aspergillus terreus* is a fungus efficient in secretion of commercial enzymes such as cellulases, xylanases and phytases. In the context of biomass saccharification, lignocellulolytic enzyme secretion was analyzed in a strain of *A. terreus* following liquid culture with sugarcane bagasse (SB) (1% *w/v*) and soybean hulls (SH) (1% *w/v*) as sole carbon source, in comparison to glucose (G) (1% *w/v*). Analysis of the fungal secretome revealed a maximum of 1.017 UI.mL^–1^ xylanases after growth in minimal medium with SB, and 1.019 UI.mL^–1^ after incubation with SH as carbon source. The fungal transcriptome was characterized on SB and SH, with gene expression examined in comparison to equivalent growth on G as carbon source. Over 8000 genes were identified, including numerous encoding enzymes and transcription factors involved in the degradation of the plant cell wall, with significant expression modulation according to carbon source. Eighty-nine carbohydrate-active enzyme (CAZyme)-encoding genes were identified following growth on SB, of which 77 were differentially expressed. These comprised 78% glycoside hydrolases, 8% carbohydrate esterases, 2.5% polysaccharide lyases, and 11.5% auxiliary activities. Analysis of the glycoside hydrolase family revealed significant up-regulation for genes encoding 25 different GH family proteins, with predominance for families GH3, 5, 7, 10, and 43. For SH, from a total of 91 CAZyme-encoding genes, 83 were also significantly up-regulated in comparison to G. These comprised 80% glycoside hydrolases, 7% carbohydrate esterases, 5% polysaccharide lyases, 7% auxiliary activities (AA), and 1% glycosyltransferases. Similarly, within the glycoside hydrolases, significant up-regulation was observed for genes encoding 26 different GH family proteins, with predominance again for families GH3, 5, 10, 31, and 43. *A. terreus* is a promising species for production of enzymes involved in the degradation of plant biomass. Given that this fungus is also able to produce thermophilic enzymes, this first global analysis of the transcriptome following cultivation on lignocellulosic carbon sources offers considerable potential for the application of candidate genes in biorefinery applications.

## Introduction

Biorefining can be defined as the sustainable processing of biomass into a spectrum of bio-products and bioenergy through a holistic approach that considers sustainability at the environmental, social and economic level (Jungmeier et al., 2013, 2014). In lignocellulosic feedstock biorefining, plant biomass is pre-treated to release cellulose, hemicellulose and lignin intermediates, with the first two of these polymers then bio-converted into fermentable pentose and hexoses, which can subsequently be further processed through sugar fermentation into bioenergy or other value-added bio-products. Lignin can also be employed in the production of chemical commodities and polymers (Windeisen and Wegener, 2012; de Jong and Gosselink, 2014). Given the global abundance of agricultural plant biomass residues, there is considerable potential today in such biorefinery-based applications in the production, amongst others, of bioethanol, industrial enzymes, organic acids, amino acids, secondary metabolites, pharmaceutical products, food, and animal feed (Silva et al., 2017).

The plant cell wall is recalcitrant to breakdown due to the complexity and heterogeneity of the diverse polymer components, each with different physical, chemical and functional properties (Alvira et al., 2010; Buckeridge and Souza, 2014; Zeng et al., 2017). Different macromolecular polymers make up plant lignocellulosic biomass, mainly comprising a complex matrix of interlinked cellulose, hemicelluloses, lignin and pectin. Although the amount of each polymer will vary according to each plant species, cellulose is typically the most abundant homopolymer in plant cell walls (40–55%), composed of glucose monosaccharides in β-1, 4 format that form a final amorphous or crystalline microfiber structure (Kimura et al., 1999; Mckendry, 2002). Hemicelluloses, while less abundant than cellulose (15–25%), still represent the second most abundant polymer on earth (Saha, 2003). The heteropolymers comprise xylans, xyloglucans, mannans, glucomannans, galactomannans, glucuronoarabinoxylans, glucuronoxylans, amongst other polysaccharides, interacting directly with cellulose fibers (Mohnen and Hahn, 1993). Lignin, whilst less abundant than cellulose (10–20%), provides structural integrity and overall stiffness to stems and root tissues (Albersheim et al., 2010). This chemically complex recalcitrant non-carbohydrate aromatic heteropolymer is made up of different syringyl, guaiacyl, and p-hydroxyphenyl monomer units (Boerjan et al., 2003). Pectins, which are also less abundant in the plant cell wall (up to 30%), are composed of highly variable polysaccharides, with a backbone composed principally of galacturonic acid with α-(1,4) bonds, together with different pectic domains of the structural classes homogalacturonan, xylogalacturonan, apiogalacturonan, arabinan, galactan, arabinogalactan I and II, and rhamnogalacturonan I and II (Ridley et al., 2001; Caffall and Mohnen, 2009; Gawkowska et al., 2018).

The enzymatic hydrolysis of plant lignocellulosic biomass is currently regarded as the most efficient biomass conversion method (Silva et al., 2017). Here, enzyme consortia with different specificities are required for lignocellulose breakdown, including cellulases, hemi-cellulases, ligninases, lytic polysaccharide mono-oxygenases (LPMOs) and cellobiose dehydrogenases (CDHs), together with the action of proteins such as swollenins and expansins (Van Dyk and Pletschke, 2012). Whilst such hydrolysis of plant cell wall polysaccharides to simple monomers has been a subject of considerable study, improved cost efficiency of enzymatic hydrolysis is essential for enzyme-based lignocellulosic biorefineries (Maijala et al., 2012; Silva et al., 2017).

Carbohydrate-active enzymes (CAZy enzymes/CAZymes) are classified as enzymes involved in the assembly, modification and breakdown of polysaccharides through their action on glycosidic bonds. As such, many are involved in the biosynthesis and degradation of the plant cell wall. These are today classified into several hundred enzyme protein superfamilies, families, and subfamilies, based on protein sequence similarity and predicted protein folding structure (Levasseur et al., 2013; Lombard et al., 2013). These broadly comprise the superfamilies of Glycosyl transferases (GT), Glycoside hydrolases (GH), Carbohydrate esterases (CE), Polysaccharide lyases (PL), and Auxiliary Activities (AA). Only GTs are involved in the formation of glycosidic bonds and assembly of complex carbohydrates, with all other superfamilies responsible for carbohydrate breakdown or modification. The GHs are responsible for the hydrolysis or modification of glycosidic bonds, the CEs for hydrolysis of carbohydrate esters, and the PLs for non-hydrolytic cleavage of glycosidic bonds. The broad AA superfamily groups redox enzymes and lytic polysaccharide mono-oxygenases (LPMOs) with different catalytic reaction mechanisms. These CAZymes are involved in both lignin breakdown and in facilitating access to plant cell wall carbohydrates for GH, CEs, and PLs (Cantarel et al., 2009; Levasseur et al., 2013). Different families of non-catalytic carbohydrate-binding modules (CBMs) can also occur within larger CAZyme protein sequences, or occasionally independently, where specific amino acid sequence domain folds enable carbohydrate-binding activities that serve to enhance the activity of the enzymatic modules.

Microbial enzymes can be employed in the bioconversion of complex lignocellulosic biomass, in hydrolysis and saccharification steps for release of simple sugars, and during subsequent fermentation into further industrial products. Bioconversion processes can be separate or can occur simultaneously, with consolidated bioprocessing technologies offering complete enzyme production and secretion, hydrolysis and fermentation bioprocesses performed by a single microorganism.

Many different prokaryotic (Palazzolo and Kurina-Sanz, 2016) and eukaryotic microorganisms are important reservoirs of plant cell wall degrading enzymes appropriate for biorefinery applications. Of these, saprophytic filamentous fungi have been more widely studied as biodegraders of lignocellulosic biomass. Today a limited number of Ascomycete species within genera such as *Trichoderma, Aspergillus, Penicillium, Neurospora, Acremonium*, and *Chaetomium*, amongst others, play important roles as sources of commercial CAZymes, preferentially for cellulose and hemicellulose degradation (Martinez et al., 2008; Ribeiro et al., 2012; Glass et al., 2013; Horta et al., 2014; Borin et al., 2015, 2017; Kameshwar et al., 2019). Basidiomycete white-rot fungi can also serve as sources of enzymes to degrade lignin, through the action of oxidative ligninolytic enzymes (Ruiz-Dueñas et al., 2013; Kameshwar and Qin, 2018). Despite the considerable advances in enzyme characterization in recent years, further bioprospection is required for microbial enzymes with increased activity, resistant to inhibitors, and appropriate for universal enzyme cocktails and enzymatic consortia.

Whilst there has been considerable investigation into secreted fungal enzymes involved in the degradation of lignocellulose, the identification of the responsible genes encoding such CAZymes and their regulation mechanisms has been more limited, despite the importance of such information in optimization of biotechnological processes (Horta et al., 2014). Whole genome analysis has progressed considerably, with the MycoCosm database now housing thousands of complete fungal genomes, with annotation data providing catalogs of genes that enable filtering for gene repertoires involved in lignocellulose degradation (Grigoriev et al., 2014). As fungal enzyme production is subject to gene induction and repression based on the carbon source (Cantarel et al., 2009), the employment of different lignocellulosic materials can serve as a promising strategy for the induction of complex cellulase and hemicellulase enzymes in filamentous fungi (Sorensen et al., 2011). Although gene expression regulation mechanisms in response to specific polysaccharides has been closely studied in model fungi (e.g., Amore et al., 2013), analyses of fungal transcriptomes on different lignocellulosic carbon sources can also reveal important information on genes encoding CAZymes, their regulation, and ultimately, the molecular mechanisms of enzymatic breakdown of natural complex lignocellulosic biomasses (Hori et al., 2014; Cragg et al., 2015; Rosnow et al., 2017).

The genus *Aspergillus* comprises more than 300 recognized species, with a number of species of importance in a variety of areas of biotechnology and food production. A number of these species are also efficient in secretion of hydrolytic enzymatic of importance for enzyme-based lignocellulosic biorefineries, with transcriptome analyses in recent years providing important information on gene expression and regulation mechanisms for genes encoding different cellulases, endoxylanases, β-xylosidases and pectinases during degradation of lignocellulosic biomass. *Aspergillus terreus* is frequently isolated from soil rhizospheres and decaying agricultural cellulosic materials in tropical and subtropical regions (Wijeratne et al., 2003; Palonen et al., 2017). In addition to producing different statins that have been widely used in the treatment of heart disease, this species also produces important commercial enzymes employed in bioprocesses that include cellulases, xylanases, lipases and phytases (Yadav et al., 1998; Varga et al., 2005; Chantasingh et al., 2006; Narra et al., 2012; Sharma et al., 2014; Pierce et al., 2017). The reference genome sequence of *A. terreus* NIH2624 shows an estimated size 29.3 Mb, with 10406 predicted gene models (Arnaud et al., 2012). Of these, a total of 477 are predicted to be related to polysaccharide hydrolysis, when compared against the Carbohydrate-Active Enzymes Database^1^. Subsequent resequencing of different strains has reported approximately 500 genes as predicted to be involved in complex polysaccharide degradation (Dadheech et al., 2019).

Analysis of gene expression in lignocellulolytic fungal species cultivated on different plant biomass substrates as sole carbon source enables identification of conserved and specific mechanisms employed in the microbial degradation of diverse plant cell wall residues (Roche et al., 2014). In this study, we examine differential expression of CAZyme-encoding genes through transcriptome profiling of *A. terreus* strain BLU24 after growth on sugarcane bagasse and soybean hulls, which are both considered promising lignocellulosic substrates for biorefinery applications, in terms of the high concentration of carbohydrates that can be converted into fermentable sugar residues (Cheng and Zhu, 2013). This fungal strain has previously been reported to efficiently secrete xylanases on lignocellulosic biomass residues (de Souza Moreira et al., 2013). Increased understanding of plant cell wall degradation mechanisms will contribute to downstream rational engineering of improved microorganisms for efficient enzymatic degradation of agricultural plant biomass residues.

## Materials and Methods

### Fungal Strain Identification

*Aspergillus terreus* strain BLU24, belonging to the fungal culture collection of the Enzymology Laboratory at the University of Brasilia (genetic heritage number 010237/2015-1), was originally isolated into pure culture from natural cotton fiber composting waste (Siqueira et al., 2009). For molecular identification confirmation, the fungus was grown on liquid Czapek Yeast Autolyzate medium (CYA) (Pitt and Hocking, 2009) for 3 days at 28°C with agitation at 120 rpm. Mycelium was washed with sterile distilled water, vacuum filtrated and lyophilized. Total DNA was extracted from 50 mg of macerated mycelium according to Raeder and Broda (1985). Quantification of total DNA was performed by comparison with a Low DNA Mass Ladder^®^ (Invitrogen), following electrophoresis in 1% agarose gels containing ethidium bromide (1.0 μg mL^–1^) and visualization under UV at 254 nm. The ribosomal DNA (rDNA) internal transcribed spacer (ITS) regions 1 and 2, together with regions of the β-tubulin and calmodulin genes, were PCR-amplified, respectively, using universal primers ITS5 and ITS4 (White et al., 1990), Bt2a and Bt2b (Glass and Donaldson, 1995), and Cmd5 and Cmd6 (Hong et al., 2006). Each PCR reaction contained 15 ng of template DNA, 0.4 μM of each primer, 200 μM dNTPs, 1.5 mM MgCl_2_, 1.0U Taq DNA polymerase and 1× IB Taq polymerase buffer (Phoneutria, Belo Horizonte, Brazil). Temperature cycling followed a program of denaturation at 95°C for 4 min, 30 cycles of denaturation at 94°C for 1 min, primer annealing at 50°C (ITS) or 60°C (β-tubulin and calmodulin genes) for 1 min, and extension at 72°C for 1 min. A final elongation period at 72°C for 5 min was included. PCR products were purified using ExoSAP-IT^®^ (USB, Cleveland, OH, United States) and forward and reverse- sequenced using the Big Dye^®^ Terminator v3.1 Cycle Sequencing kit (Applied Biosystems, Foster City, CA, United States). Products were separated on an ABI 3130xl DNA sequencer (Applied Biosystems, Foster City, CA, United States). Generated sequences were quality filtered and clustered into contigs using the program Sequencher, version 4.8 (Gene codes Corporation, Ann Arbor, MI, United States). Molecular-based identification was performed on the basis of sequence identity using the program BLASTn (Altschul et al., 1997), against the NCBI nucleotide nr database. The ribosomal DNA ITS, β-tubulin and calmodulin gene consensus sequences confirming species level identification were deposited in GenBank under the accession number MT461408, MT472459, and MT472460, respectively.

### Experimental Design

*Aspergillus terreus* BLU24 was grown in liquid minimal medium [7 g KH_2_PO_4_, 2g K_2_HPO_4_, 0.5 g MgSO_4_, 1.6 g de (NH_4_)_2_ SO_4,_ pH 7.0, per L of distilled water], supplemented with sugarcane bagasse (SB), soybean hulls (SH) or glucose (G) (Sigma Aldrich) as sole carbon source (each at 1% w/v). Each plant biomass residue-derived carbon source, obtained from local sources, was pretreated through the addition of a 1:1 volume of water, autoclaving for 2 h at 120 psi, washing with distilled water, drying at 60°C for 48 h and material milling. For elimination of reducing sugars, these carbon sources were washed with deionized water, with reducing sugar elimination detected using the colorimetric dinitrosalicylic acid (DNS) assay (Miller, 1959). Fungal spores were harvested from a fresh culture of *A. terreus* BLU24 grown on Potato Dextrose Agar medium (Thermo Fisher Scientific, Paisley, United Kingdom), adjusted to a concentration of 1 × 10^8^ conidiospores mL^–1^ and employed as inoculum. Liquid cultures were grown in 100 mL of each growth media in 250 mL Erlenmeyer flasks at 28°C and 128 rpm over a 9-day period. Culture arrangement followed a randomized block design, with three replicates for each growth treatment and incubation time point. Treatments were labeled according to the following codes: sugarcane carbon source, 36 h incubation (SB36); soybean hull carbon source, 36 h incubation (SH36); glucose carbon source, 36 h incubation (G36); sugarcane carbon source, 48 h incubation (SB48); soybean hull carbon source, 48 h incubation (SH48); glucose carbon source, 48 h incubation (G48).

### Enzyme Analysis

Hydrolytic enzyme activities were evaluated during 9 days at 24 h intervals. Xylanase, CMCase, pectinase, and FPase assays were conducted using the DNS assay at pH 5.0 (Miller, 1959). Each assay, conducted on ELISA microtiter plates, comprised 10 μl of the fungal secretome, together with xylan (1% wt/vol), carboxy methylcellulose (1% wt/vol), or pectin (1% wt/vol) as substrate. Reactions were incubated for 30 min at 50°C. Quantification of total cellulases was performed with an assay for FPase activity according to Ghose (1987). 10 μl of each secretome was added to Whatman^®^ filter paper No. 1.0 × 0.6 cm followed by incubation for 1 h at 50°C. All assays were conducted in triplicate. Reducing sugars from each assay were quantified on a spectrophotometer at a wavelength of 540 nm (Spectramax plus 384, Molecular Devices), with calibration using standard curves of glucose, xylose, mannose and galacturonic acid. Absorbance values obtained were expressed as international units (UI). The Tukey’s test was performed at a *P* < 0.05 significance level for comparison of datasets on different carbon sources.

### Scanning Electron Microscopy

The degradation of each lignocellulosic carbon source was examined by scanning electron microscopy (SEM) following growth of *A. terreus* BLU24 for 36 and 48 h in liquid minimal medium supplemented with SB or SH. Culture filtrates were washed using Karnovsky buffer (0.05M; pH 7.2) and material fixed for 4 h in 0.05M cacodylate buffer at pH 7.4. Samples were dehydrated using acetone and postfixed with 1% osmium tetroxide for 1 h. Following washes in liquid CO_2_ at 4°C, exposure to critical point drying (Emitech K850, Kent, United Kingdom) and mounting on copper stubs for sputter coating with 20 nm gold particles, samples were observed at different magnifications using a Zeiss DSM 962 scanning electron microscope.

### Total RNA Extraction

Conidiospores of *A. terreus* BLU24 were inoculated as described earlier into 100 mL minimal medium containing 1% glucose (w/v) or 1% of each crop-derived carbon source. Cultures were incubated on an orbital shaker at 128 rpm at 28°C for 36 or 48 h. All treatments (carbon source + growth period) were performed with repetition, with three biological replicate samples per treatment and duplicate bioassays. Culture mycelia were harvested after each treatment growth period and immediately frozen in liquid nitrogen. Isolation of total RNA from frozen mycelia was performed according to Brasileiro and Carneiro (2015), with DNA removed using Ambion^®^ DNA-free^TM^ DNase Treatment and Removal Reagents (Thermo Fisher Scientific, Waltham, MA, United States). RNA concentration and integrity were estimated with a NanoDrop 2000c spectrophotometer (Thermo Scientific, Waltham, MA, United States) with a quality cut-off value of 1.8 employed for the A260:280 ratio. Integrity of RNA was also measured using an Agilent 2100 Bioanalyzer (Agilent Technologies, Palo Alto, CA, United States).

### Construction of cDNA Libraries and RNA-Seq Transcriptome Profiling

A total of 12 RNA samples, representing all treatments and biological replicates, were shipped in RNAstable (Biomatrica) according to the manufacturer’s instructions. Messenger RNA isolation, cDNA library preparation and Illumina RNA-Seq were performed by Eurofins MWG Operon (Alabama, United States) from 10 μg of each total RNA sample. cDNA libraries were prepared using the TruSeq Sample RNA preparation kit v3 Kit according to the manufacturer’s specifications and paired-end-sequenced (2x 100 base pairs) in two flowcell lanes on an Illumina Hiseq2500 sequencer (Illumina Inc., San Diego, CA, United States).

### Sequence Processing and Bioinformatic Analyses

Sequence reads from each cDNA library were adapter-trimmed using the program Trimmomatic (Bolger et al., 2014) and quality assessment conducted using ea-utils (Aronesty, 2011). High quality reads (Fastq QC > 30) were mapped to the reference genome sequence for *A. terreus* strain NIH 2624^2^ using the program Novoalign/Useq^3^. Unmapped sequence reads were analyzed through alignment to the NCBI non-redundant protein sequence database (nr) using Diamond (Buchfink et al., 2015), with an *E*-value cut-off at 10^–9^. Reads accurately mapped to individual genes in the reference genome were counted using HTseq-count (Anders et al., 2015), with differences in gene expression between evaluated treatments determined using DESeq (Anders and Huber, 2010). Statistically significant differentially expressed genes (DEGs) were considered if a log2 fold change (FC) was at least ≥2-fold and at a probability level of *p* ≤ 0.01.

Sequences were functionally annotated according to Gene Ontology (GO) terms (Ashburner et al., 2000), with over- and under-representation of DEGs assigned to GO terms analyzed using the program FUNC (Prüfer et al., 2007) and category term redundancy eliminated using the program REVIGO^4^.

Genes encoding hydrolytic enzymes glycoside hydrolases, glycosyltransferases, carbohydrate-binding modules and carbohydrate esterases were identified through gene sequence alignment against the updated Carbohydrate-Active Enzymes (CAZymes) database (see text footnote 1) (Cantarel et al., 2009). Annotation validation at the family and subfamily level was conducted using CUPP for analysis of predicted conserved unique octamer peptide signatures (Barrett and Lange, 2019; Barrett et al., 2020).

### Quantitative Real-Time PCR Validation of RNA-Seq-Derived Gene Expression Data

Validation of expression of 8 selected DEGs identified based on *in silico* transcriptome data was conducted by quantitative real-time PCR (RT-qPCR) analysis. Thermocycling was conducted on a Step One Plus Real Time PCR System (Applied Biosystems) using a Platinum^®^ SYBR^®^ Green qPCR Super Mix-UDG w/ROX kit (Invitrogen, Carlsbad, CA, United States) according to the manufacturer’s guidelines and 1 μL of template cDNA. Specific primers were designed with Primer Express^®^ (Applied Biosystems) and evaluated for specificity at OligoAnalyzer 3.1 – IDT^5^. All cDNA libraries were synthesized using original RNA samples that were employed for RNA-Seq analysis. Three independent biological replicates were analyzed for each treatment and three technical replicates included per amplification. Total RNA was initially treated with 2 U of Amplification Grade DNase I (Invitrogen, Carlsbad, CA, United States) and cDNA then synthesized using Oligo(dT)20 primers (Invitrogen, Carlsbad, CA, United States) and SuperScript^®^ II Reverse Transcriptase (Invitrogen, Carlsbad, CA, United States). Thermal cycling was conducted by 40 cycles of denaturation at 95°C for 15 s, followed by primer annealing and amplicon extension at 60°C for 30 s. *A. terreus* Actin and rDNA 18S genes were included as stable reference genes for normalization. Cycle threshold (Ct) values were calculated using the program SDS 2.2.2 (Applied Biosystems, Foster City, United States), with PCR product specificity determined according to the Tm (dissociation) of the amplification product. Individual amplification efficiencies were calculated with the program LinRegPCR, version 2013.0 and gene expression values calculated according to the 2-ΔΔCT method (Livak and Schmittgen, 2001).

## Results

### Scanning Electron Microscopy Analysis

**FIGURE 1:**
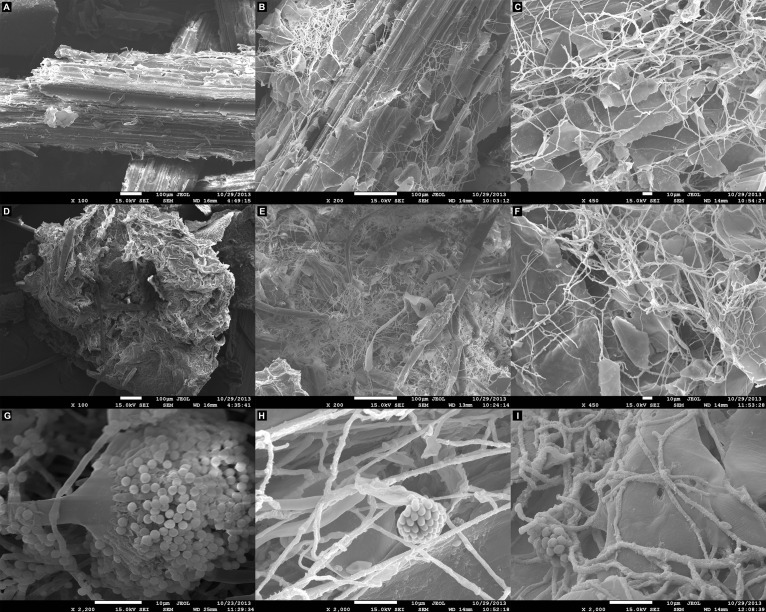
Scanning electron microscopy (SEM) analysis of the degradation of lignocellulosic carbon sources following growth of *Aspergillus terreus* BLU24 in liquid minimal medium supplemented with each carbon source. **(A)** Non-inoculated pre-treated sugarcane bagasse parenchyma in liquid minimal medium culture. **(B)** Inoculated pre-treated sugarcane bagasse in liquid minimal medium culture after 36 h incubation, 200X magnification. **(C)** Inoculated pre-treated sugarcane bagasse in liquid minimal medium culture after 48 h incubation, 450X magnification. **(D)** Non-inoculated pre-treated soybean hull parenchyma in liquid minimal medium culture. **(E)** Inoculated pre-treated soybean hull in liquid minimal medium culture after 36 h incubation, 200X magnification. **(F)** Inoculated pre-treated soybean hull in liquid minimal medium culture after 48 h incubation, 450X magnification. **(G)** *A. terreus* BLU24 conidiophore and conidiospore development, 2200X magnification. **(H)** *A. terreus* BLU24 conidiospores and hyphae on pre-treated sugarcane bagasse in liquid minimal medium culture after 48 h incubation, 2000X magnification. **(I)** *A. terreus* BLU24 conidiospores and hyphae on pre-treated soybean hull in liquid minimal medium culture after 48 h incubation, 2000X magnification.

Scanning electron microscopy observations following growth of *A. terreus* BLU24 in liquid minimal medium supplemented with the lignocellulosic carbon sources SB and SH showed parenchyma surface disruption and fungal colonization on each after 36 h, with considerable hyphal growth and conidiospore production visible after 48 h (Figure 1).

### Enzyme Activity Profiles on Lignocellulosic Biomass

**FIGURE 2:**
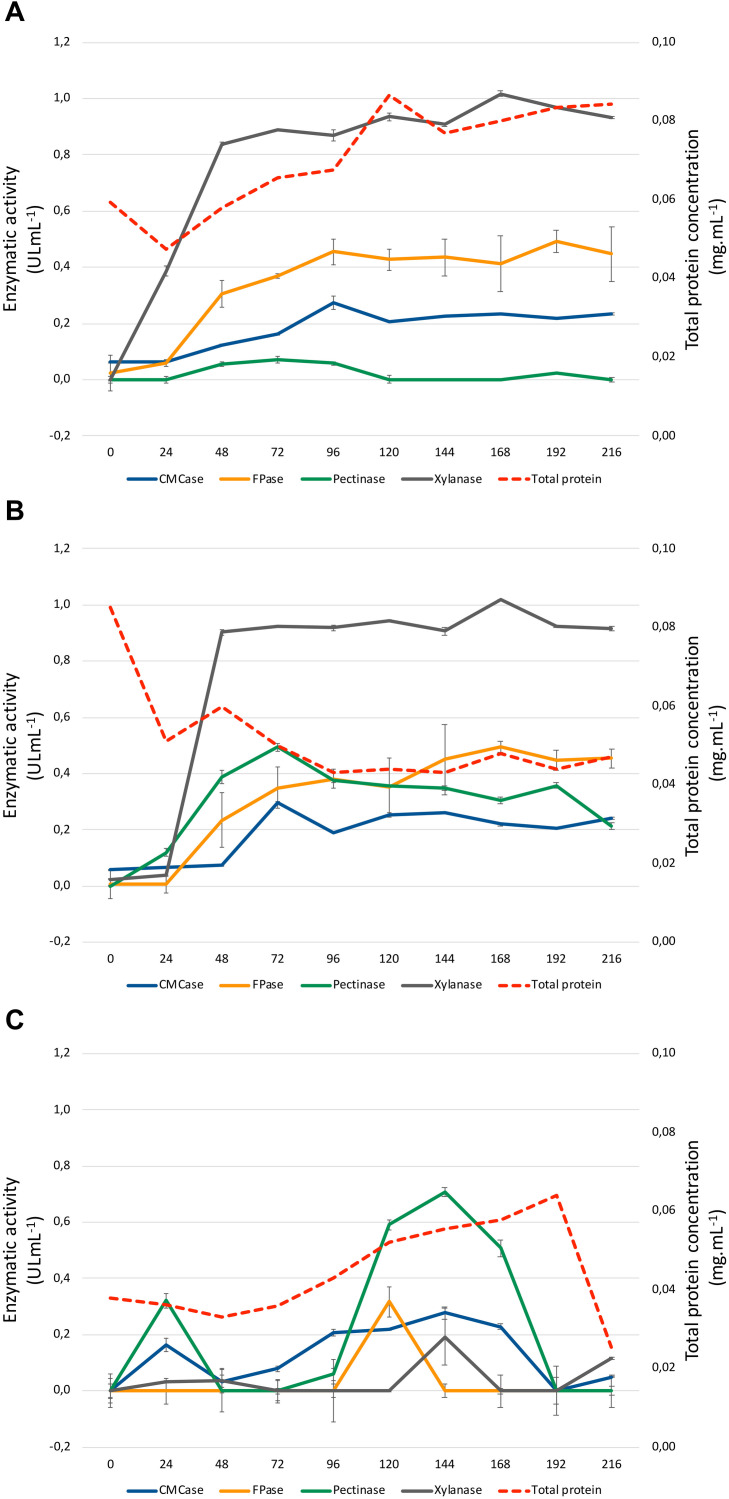
Enzymatic activities of *Aspergillus terreus* BLU24 following growth in liquid minimal medium supplemented with sugarcane (1% w/v) **(A)**, soybean hull (1% w/v) **(B)**, or glucose (1% w/v) **(C)**, as sole carbon source. Enzyme activities in response to xylan, carboxymethylcellulose, pectin, and filter paper substrate were determined through a DNS assay at pH 5.0.

Enzymatic profiles of *A. terreus* BLU24 were evaluated over a 9-day period following growth on liquid minimal medium culture supplemented with SB or SH as sole lignocellulosic carbon source. Enzymatic secretions were compared to those observed following growth on the same minimal medium with G as sole carbon source, employed as a control treatment. Growth on both lignocellulosic carbon sources resulted in a rapid increase in xylanases during the first 48 h [(0.839 ± 0.007; 0.903 ± 0.010 UI mL^–1^), respectively for SB and SH], remaining fairly constant for the remainder of the investigated time period (Figure 2). This was in contrast to growth on G, where xylanase activities remained low at 48 h (0.038 ± 0.040X UI mL^–1^). Activities for CMCases, pectinases and FPases were comparatively lower than observed for xylanases after 48 h (0.124 ± 0.001, 0.072 ± 0.002; 0.055 ± 0.009, 0.388 ± 0.025; 0.304 ± 0.048, 0.234 ± 0.098 UI mL^–1^), respectively on SB and SH, although again considerably raised in comparison to values following growth on G as carbon source at 48 h. Once again, activities remained fairly constant for these enzymes throughout the remaining time period of growth investigated. Considering the kinetics observed for xylanases on SB and SH, and likely evidence of carbon catabolic repression during the first 48 h of growth on G, 36 and 48 h were selected as time points for RNA extraction and subsequent analysis of the transcriptome of *A. terreus* BLU24 following growth on SB or SH lignocellulosic carbon sources in comparison to G.

### Illumina Hiseq 2500 Statistics

Illumina Hiseq 2500 sequencing of cDNA libraries yielded, for bioassay 1, after adapter trimming, a total of 317 million reads for the multiplexed data, totaling 31.73 Gb of data. Similar numbers were observed for bioassay 2, with 335 million reads, and 33.59 Gb of data. Percentages of high-quality sequences (Fastq QC > 30) were similar for all treatments and repeat bioassays, averaging 81.42% after adapter trimming (Supplementary Table 1). All Illumina RNA-Seq datasets can be found in the NCBI Sequence Read Archive (SRA) database (BioProject ID PRJNA631730).

### Differential Gene Expression

The reference genome for *A. terreus* (NIH 2624, BioProject ID PRJNA15631) contains a total of 10406 gene models. Alignment of the sequence reads generated for each cDNA library revealed accurate mapping to over 8000 gene models throughout, indicative of high-quality mRNA for all the biological treatments and replicates. Annotation data for unmapped sequence reads aligned to the NCBI nr database are summarized in Supplementary Table 2. Specifically, for mapped reads, a total 8812 genes were expressed in *A. terreus* BLU24 during growth on G as carbon source, with 8837 expressed following growth on SB and 8799 on SH.

**FIGURE 3:**
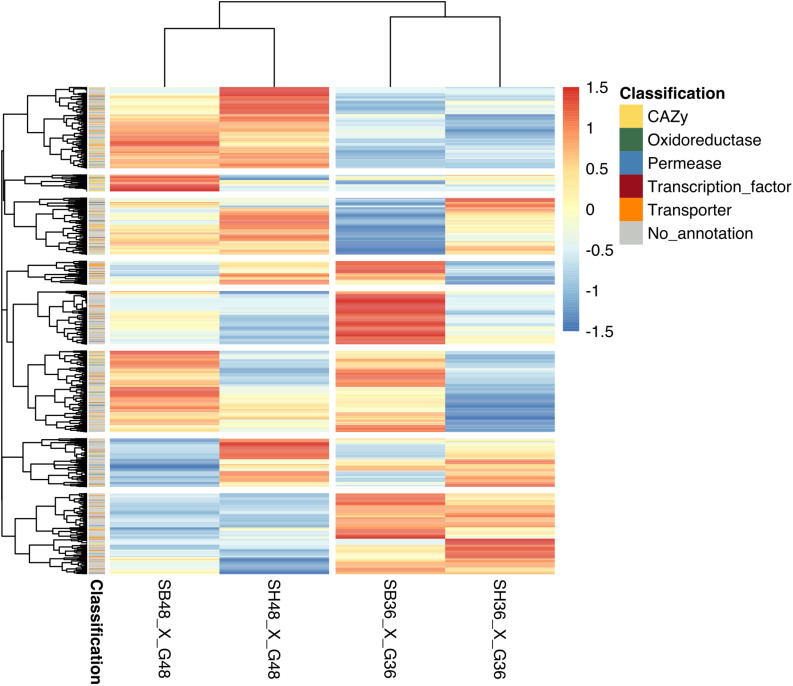
Heatmap depiction of hierarchically clustered groups of *Aspergillus terreus* BLU24 genes according to gene expression modulation following growth on different carbon sources. Gene expression modulation was compared between the growth treatments SB36 and G36, SB48 and G48, SH36 and G36, and SH48 and G48. Statistically significant differentially expressed genes were considered if a log2 fold change (FC) was at least ≥2-fold and at a probability level of *p* ≤ 0.01. All FC values below –6 or above 6 were considered as minimum or maximum values, respectively.

Differential expression in genes was calculated based upon comparison of read counts mapped to a particular reference genome gene model between treatments. Datasets were compared between growth on each lignocellulosic carbon source (SB or SH) versus growth on equivalent glucose controls. Considering analysis parameters of log2 FC at least ≥2-fold and at a probability level of *p* ≤ 0.01, considerable modulation of gene expression in the fungus was apparent, with statistically significant DEGs identified according to growth on each lignocellulosic carbon source, as well as growth period. Global analysis revealed similarities in gene expression modulation patterns according to growth period for both SB and SH, with numerous CAZyme-, oxidoreductase-, permease-, transcription factor- and transporter-encoding genes amongst the DEGs (Figure 3). In total, 642 DEGs were identified following growth on SB for 36 h, with 372 up-regulated, and 270 down-regulated. At 48 h, 750 DEGs were observed, with 414 up-regulated and 336 down-regulated. In the case of SH as sole carbon source, at 36 h a total of 648 DEGs were identified, with 300 up-regulated and 348 genes down-regulated. At 48 h, 856 DEGs were observed, with 479 up-regulated and 377 down-regulated.

### Gene Ontology-Based Representation of Differentially Expressed Genes

Gene ontology categories were assigned to DEGs using FUNC to identify over or under-representation of GO terms, with enrichment values compared with those for all genes present in the *A. terreus* NIH 2624 reference genome. With a total of 13 different enriched categories, considerable enrichment was observed for terms related to lignocellulose hydrolysis for all treatments for *A. terreus* BLU24 on SB and SH when compared to expected distribution in the reference genome (Supplementary Figure 1). Significant enriched categories related to the focus of the study where genes were up-regulated on the lignocellulosic carbon sources in comparison with glucose comprised carbohydrate binding, starch binding, catalytic activity, oxidoreductase activity, carboxylic ester hydrolase activity, hydrolase activity, hydrolyzing O-glycosyl compounds, and carbon-sulfur lyase activity. In the case of SB, enrichment was only observed after 48 h, for the categories of carbohydrate binding, starch binding, oxidoreductase activity and carboxylic ester hydrolase activity. SH displayed fold-enrichment for a greater number of the relevant terms than SB, with exclusive gene enrichment for upregulated genes in the categories of catalytic activity, hydrolase activity, hydrolyzing O-glycosyl compounds, and carbon-sulfur lyase activity. On this carbon source, exclusive enrichment was also observed at 36 h for the categories of carbohydrate binding and starch binding. Enriched GO terms for DEGs up-regulated during growth on G as sole carbon source, by contrast, comprised heme binding, cofactor binding, catalytic activity, tryptophan synthase activity and transmembrane transport.

### Expression of Cazyme-Encoding Genes

The reference *A. terreus* NIH 2624 reference genome contains a total of 580 predicted CAZyme genes, classified in different families, with 475 potentially involved in carbohydrate hydrolysis. In this study, a total of 89 expressed CAZyme-encoding genes were identified in the *A. terreus* BLU24 transcriptome following growth on SB. A total of 29 genes encoding glycoside hydrolases were expressed and likely involved in the depolymerization of cellulose, together with a further 23 glycoside hydrolase genes likely involved in the degradation of hemicellulose. A further three genes encoding CE family proteins, two genes encoding CBM family proteins and five genes encoding the LPMO AA9 family proteins were also expressed during growth on SB (Supplementary Table 2). In the case of SH, for 91 CAZyme-encoding genes, 35 were expressed that encode glycoside hydrolases potentially involved in the depolymerization of cellulose, together with 28 glycoside hydrolase genes likely involved in hemicellulose degradation. Additionally, genes encoding one CE family protein, two PL family proteins, two CBM family proteins, two GT family proteins, six LPMO AA9 family proteins, and three genes encoding GH28, GH77, and GH88 proteins associated with pectin degradation, were also expressed on this carbon source (Supplementary Table 2).

**FIGURE 4:**
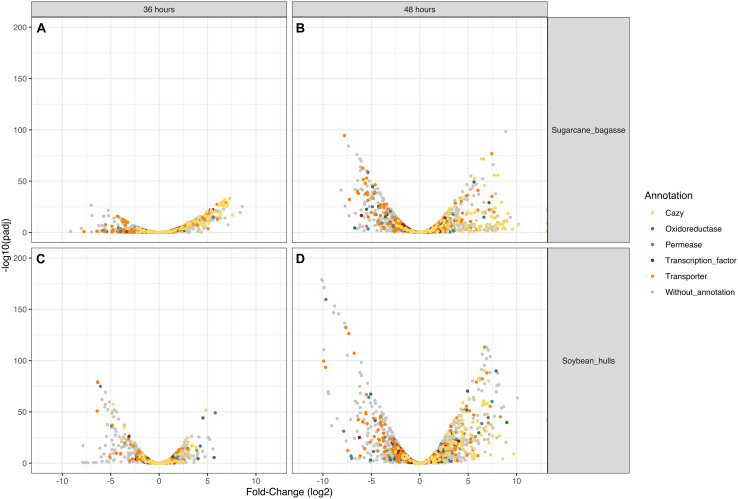
Volcano plot analysis of differential gene expression in *Aspergillus terreus* BLU24 according to carbon source and incubation time period. **(A)** SB36 vs. G36; **(B)** SB48 vs. G48; **(C)** SH36 vs. G36; **(D)** SH48 vs. G48. Genes that were highly modulated, in relation to equivalent treatments with glucose as sole carbon source, appear further to the left and right sides of the plot, with highly significant changes in expression located higher on the plot. Yellow dots: CAZyme-encoding genes (all families); Red dots: genes encoding transcription factors; Green dots: genes encoding permeases; Blue dots: genes encoding oxidoreductases; Gray dots: genes of other function.

**FIGURE 5:**
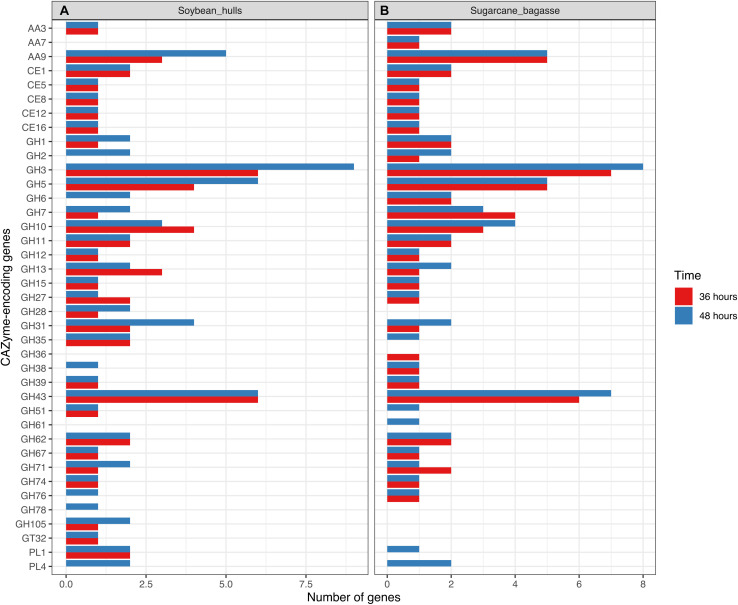
Barplot representation of genes related to cellulose, hemicellulose and pectin depolymerization in transcriptome data for *Aspergillus terreus* BLU24 following 36 and 48 hours growth on soybean hull **(A)** and sugarcane bagasse **(B)** as sole carbon source.

**FIGURE 6:**
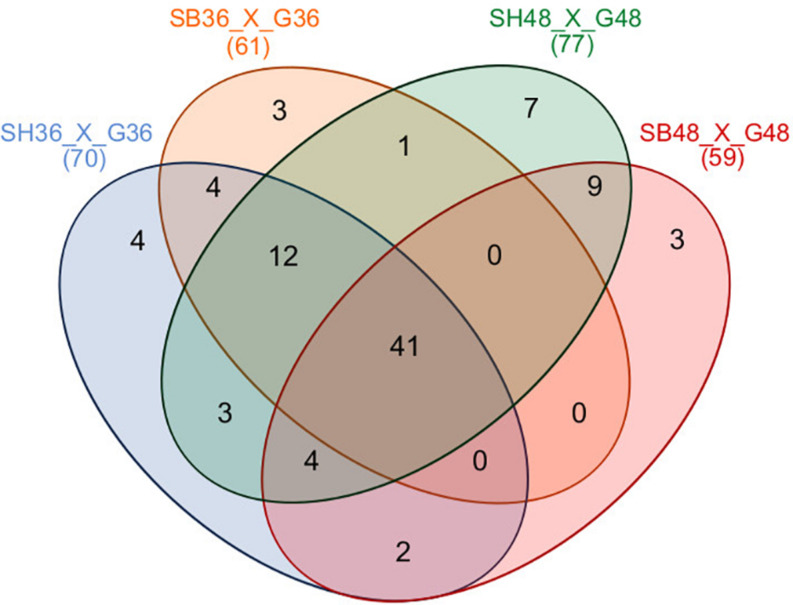
Venn diagram summary of differentially expressed CAZyme-encoding genes for *Aspergillus terreus* BLU24 among treatments SB36, SB48, SH36, and SH48. Differential gene expression was considered significant if relative expression in comparison to equivalent treatments with glucose as sole carbon source showed a log2 fold change (FC) of at least ≥2-fold, at a probability level of *p* ≤ 0.01. Overlapping regions of the diagram represent DEGs common to different growth treatments.

**FIGURE 7:**
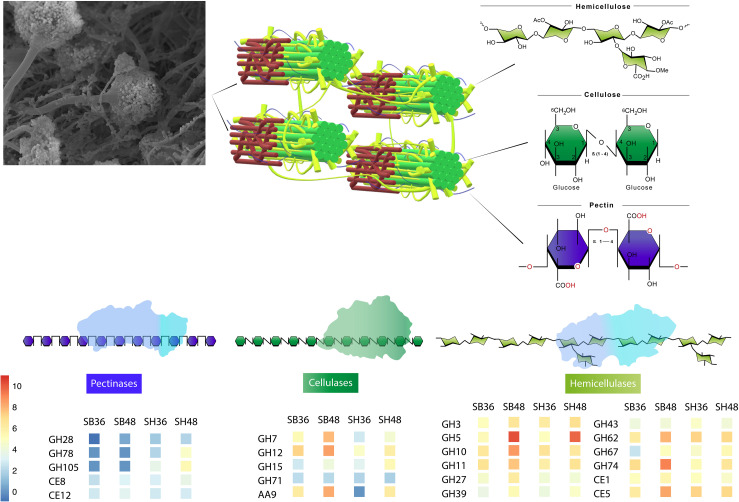
Representative model of conserved CAZyme families in *Aspergillus terreus* BLU24 involved in degradation of lignocellulosic biomasses sugarcane bagasse and soybean hull, based on genes with significant increased expression after growth on both carbon sources and at each evaluated time point, in comparison with equivalent growth on glucose.

Differential expression of CAZyme-encoding genes in treatments on SB or SH in comparison with equivalent growth periods on glucose as carbon source (log2 fold change (FC) at least ≥2-fold and at a probability level of *p* ≤ 0.01) is depicted in Figure 4 and Supplementary Figure 2. A total of 77 CUPP-validated CAZyme-encoding genes were observed to be significantly differentially up-regulated on SB, across the two growth time points investigated (Table 1 and Figure 5). These comprised approximately 78% glycoside hydrolases, 8% carbohydrate esterases, 2.5% polysaccharide lyases, and 11.5% auxiliary activities. Analysis of the glycoside hydrolase family revealed significant up-regulation for genes encoding 25 different GH family proteins, with predominance for families GH3, 5, 7, 10, and 43. For SH, a total of 83 CAZyme-encoding genes were also significantly up-regulated in comparison to G. These comprised approximately 80% glycoside hydrolases, 7% carbohydrate esterases, 5% polysaccharide lyases, 7% auxiliary activities (AA), and 1% glycosyltransferases. Similarly, within the glycoside hydrolases, significant up-regulation was observed for genes encoding 26 different GH family proteins, with predominance, as for SB, for families GH3, 5, 10, 31, and 43. Venn analysis of CAZyme-encoding DEGs across treatments SB36, SB48, SH36, and SH48 revealed DEGs exclusively up-regulated on each carbon source (Figure 6 and Table 1), as well as 41 up-regulated DEGs common to all four treatments, likely indicative of conserved function in hydrolysis of lignocellulose in *A. terreus* (Figure 7).

**TABLE 1:** Summary of CAZyme-encoding genes in *Aspergillus terreus* BLU24 with significant increased expression after growth on sugarcane bagasse and soybean hulls in comparison with growth on glucose as carbon source.

					log2 Fold Change ≥2; padj ≤ 0.01			
Class	Gene ID	Enzyme description	CAZy Family	CAZy subfamily	SB36	SB48	SH36	SH48
Cellulases	ATEG_10194	Endoglucanase	GH61/AA9			5,05		
	ATEG_00687	Beta-glucosidase 1	GH1		6,54	8,05		2,82
	ATEG_03047	Beta-glucosidase	GH3		3,24	6,35		5,59
	ATEG_07419	Beta-D-glucoside glucohydrolase	GH3		2,18	4,41		4,26
	ATEG_09314	Beta-glucosidase	GH3			4,69	3,91	4,5
	ATEG_05003	Exoglucanase I precursor	GH5	GH5_5	6,13	8,97		4,38
	ATEG_04390	Endoglucanase 3 precursor	GH5	GH5_5		4		
	ATEG_10292	Cellulase	GH5	GH5_27			5,97	
	ATEG_00193	Exoglucanase II precursor	GH6		4,62	7,38		2,7
	ATEG_07493	Exoglucanase 2 precursor	GH6		6,59	8,54		3,55
	ATEG_05002	Exoglucanase I precursor	GH7		6,33	8,59		3,58
	ATEG_08705	Endoglucanase EG-1 precursor	GH7		5,4	7,72		
	ATEG_03727	Exoglucanase I precursor	GH7		2,9	5,2	2,43	
	ATEG_08700	Endoglucanase EG-1 precursor	GH7		5,39	7,82	2,75	4,41
	ATEG_07420	Endoglucanase I precursor	GH12		7,28	8,42	4,72	6,03
	ATEG_04375	Glucoamylase precursor	GH15		2,78	4,01	3,26	5,46
	ATEG_07752	Extracellular alpha-1,3-glucanase	GH71		5,71	6,25	4,74	6,55
	ATEG_07790	Endoglucanase B	AA9		4,01	6,98	4,72	5,63
	ATEG_08942	Endoglucanase-4	AA9		5,26	7,76	4,35	5,23
	ATEG_04210	Endo-1,4-beta-glucanase	AA9		6,21	8,18	4,36	6,21
Hemicellulases	ATEG_09052	Exo-1,4-beta-xylosidase	GH3		5,14	6,49	6,32	6,95
	ATEG_09991	Endo-1,4-beta-mannanase	GH5	GH5_7	3,66	7,39	4,35	6,97
	ATEG_02669	Mannanase	GH5	GH5_7	6,27	*	6,27	*
	ATEG_08654	Endo-beta-mannanase	GH5	GH5_7	5,25	10,2	5,07	9,69
	ATEG_08906	Endo-1,4-beta-xylanase	GH10		6,78	7,88	5,03	5,79
	ATEG_00809	Endo-1,4-beta-xylanase A precursor	GH10			9,58	6,96	
	ATEG_03410	Endo-1,4-beta-xylanase precursor	GH10		6,43	8,68	6,66	6,43
	ATEG_04943	Endo-1,4-beta-xylanase B precursor	GH11		4,73	6,04	5,4	5,46
	ATEG_07461	Endo-1,4-beta-xylanase A precursor	GH11		6,79	7,61	6,1	6,74
	ATEG_03427	Alpha-galactosidase	GH27		4,55	6,14	4,23	4,27
	ATEG_08278	Alpha-glucosidase	GH31				3,33	
	ATEG_02528	Glycoside hydrolase	GH31		3,07	3,08	3,25	2,92
	ATEG_02966	Alpha-glucosidase	GH31				3,18	5,56
	ATEG_05177	Alpha-glucosidase	GH31					2,85
	ATEG_06730	Hypothetical protein similar to alpha-glucosidase (593 aa)	GH31			2,77		2,16
	ATEG_00616	Beta-galactosidase	GH35			2,84	2,63	3,58
	ATEG_01650	Mannosidase II	GH38		2,39	3,13		3,06
	ATEG_06199	Xylan 1,4-beta-xylosidase	GH39		3,77	5,33	3,91	4,69
	ATEG_01292	Arabinofuranosidase	GH43	GH43_14	4,01	4,13	4,79	4,2
	ATEG_07817	Endo-arabinase	GH43		3,68	2,99		3,57
	ATEG_10193	Xylosidase	GH43	GH43_36	4,28	5,7	4,73	5,34
	ATEG_03688	Endo-1,5-alpha-L-arabinosidase	GH43	GH43_6	6,39	6,98	6,56	7,1
	ATEG_05083	Xylosidase/arabinosidase	GH43	GH43_14	4,8	6,67	5,54	6,58
	ATEG_10072	Endo-1,4-beta-xylanase XynD	GH43	GH43_29	4,25	6,97	4,94	4,25
	ATEG_00093	Beta-xylosidase	GH43/GH117	GH43_1	3,14	4,76	4,09	4,44
	ATEG_07868	Alpha-L-arabinofuranosidase	GH51			3,06	4,39	4,3
	ATEG_10071	Alpha-L-arabinofuranosidase	GH62		6,21	7,92	7,23	7,17
	ATEG_10379	Alpha-L-arabinofuranosidase precursor	GH62		6,49	7,78	6,24	6,33
	ATEG_06085	Alpha-glucuronidase	GH67		2,54	5,16	4,57	5,51
	ATEG_04708	Endoglucanase C	GH74		6,96	9,1	5,4	6,48
	ATEG_09048	Endo mannanase	GH76		2,25			
	ATEG_09843	Acetyl xylan esterase	CE1		5,09	5,58	5,16	4,59
	ATEG_04709	Acetyl xylan esterase precursor	CE5		7,06	8,29	7,22	6,87
	ATEG_05416	Endoglucanase IV precursor	AA9		6,37	8,81		3,42
Pectinases	ATEG_07152	Exopolygalacturonase	GH28					2,02
	ATEG_04991	Endopolygalacturonase	GH28			7,82	8,45	8,55
	ATEG_02922	Alpha-L-rhamnosidase	GH78				2,05	4,75
	ATEG_07907	Unsaturated rhamnogalacturonan hydrolase	GH105				3,57	5,44
	ATEG_01704	Pectinesterase	CE8		2,41	3,08	3	3,71
	ATEG_03511	Rhamnogalacturonan acetylesterase	CE12		2,75	3,32	2,67	2,82
	ATEG_02193	Rhamnogalacturonate lyase A	PL1	PL1_4		5,15	4,15	5,22
	ATEG_07593	Pectin lyase A	PL1	PL1_4		3,03	3,22	3,15
	ATEG_10327	Rhamnogalacturonate lyase B	PL4	PL4_3			4,91	7,01
Others	ATEG_09566	Conserved hypothetical protein (633 aa)	GH76			2,72		2,73
	ATEG_04135	Hypothetical protein similar to BGLC protein (488 aa)	GH1		2,95		3,94	4,47
	ATEG_04784	Beta-D-galactosidase	GH2		2,48	3,72		2,62
	ATEG_09745	Glycoside hydrolase	GH2			2,39		2,5
	ATEG_08684	conserved hypothetical protein (1120 aa)	GH2			2,17		
	ATEG_04963	Glycoside hydrolase	GH3				5,73	Inf
	ATEG_02724	Predicted protein	GH3		5,08	6,49	5,14	6,19
	ATEG_04069	Hypothetical protein similar to Cel3d (1422 aa)	GH3			2,5		2,16
	ATEG_06617	Hypothetical protein similar to beta-glucosidase (868 aa)	GH3		4,06	3,62	3,45	3,71
	ATEG_07383	Predicted protein	GH3		7,56	8,59	8,45	8,23
	ATEG_09329	Conserved hypothetical protein (840 aa)	GH3		4,9	5,82		
	ATEG_06369	Conserved hypothetical protein (470 aa)	GH5	GH5_22	3,57	5,65	3,88	5,16
	ATEG_07190	Predicted protein	GH10		4,17	7,22	7,03	7,5
	ATEG_00838	Alpha-amylase	GH13	GH13_5	4,49	3,8	3,08	4,43
	ATEG_08279	Alpha-amylase	GH13	GH13_1			2,64	
	ATEG_02515	Hypothetical protein similar to taka-amylase (471 aa)	GH13	GH13_1			5,82	6,52
	ATEG_10103	Alpha-amylase precursor (608 aa)	GH13	GH13_1		3,74		
	ATEG_02160	Conserved hypothetical protein (642 aa)	GH27				4,35	
	ATEG_07446	Hypothetical protein similar to beta-galactosidase (907 aa)	GH35				3,21	4,26
	ATEG_07929	Alpha-galactosidase C precursor	GH36		2,63			
	ATEG_07778	Glycoside hydrolase	GH71		4,54		2,79	4,54
	ATEG_02892	Cell wall glycoside hydrolase YteR	GH105					2,41
	ATEG_01914	Hypothetical protein similar to ferulic acid esterase A	CE1		6,7	7,69	6,18	6,82
	ATEG_03676	Cellulose-binding GDSL lipase/acylhydrolase	CE16		6,15	8,99	6,04	7,03
	ATEG_08610	Predicted protein (454 aa)	PL4	PL4_5			3,63	5,97
	ATEG_09491	GMC oxidoreductase, putative	AA3	AA3_2	2,33	3,44	3,29	2,68
	ATEG_09993	Cellobiose dehydrogenase	AA3	AA3_1	5,37	7,49		
	ATEG_07698	6-hydroxy-D-nicotine oxidase	AA7		2,24	2,12		
	ATEG_05081	Fungal cellulose binding domain	AA9		4,65	6,21	5,18	5,46
	ATEG_00990	Glycosyl transferase	GT32					3,54

*Calculation not possible due to read absence in RNAseq data for corresponding treatment with glucose as carbon source.

Up-regulated CAZyme-encoding DEGs coding for GHs involved in the depolymerization of cellulose comprised genes belonging to the GH families 1, 3, 5, 6, 7, 12, 15, and 71, with exclusive expression for GH5 members in SB (ATEG 04390), and similarly in SH (ATEG 10292). Seven up-regulated genes belonging to the GH families, 7, 12, 15, and 71 were common across carbon sources and time points. Considerable up-regulation (>6-fold) in specific treatments on SB was also observed for genes belonging to GH1 (ATEG 00687), GH5 (ATEG 05003), and GH6 (ATEG 00193, 07493). With regard to hemicellulose degradation, up-regulation was observed in genes in the GH families 3, 5, 10, 11, 27, 31, 35, 38, 39, 43, 51, 62, 67, 74, and 76, as well as CE families 1 and 5. Within this category, CAZyme-encoding DEGs associated with degradation of mannan polysaccharides were observed (GH5: ATEG 02669, 08654; GH38: ATEG 01650; GH76: ATEG 09048), with the latter up-regulated exclusively on SB. DEGs related to Xylan hydrolysis included, for both carbon sources, β-xylosidases (EC 3.2.1.37) (GH3, GH43) (ATEG 09052, 06199, 10193, 00093), xylanases (EC 3.2.1.8) (GH10, 11, 43) (ATEG 08906, 00809, 03410, 04943, 07461, 10072), α-L-arabinofuranosidase (EC 3.2.1.55) (GH43) (ATEG 01292, 07868, 10071, 10379), as well as other xylan-debranching enzymes. Twenty two genes belonging to the GH families 3, 5, 10, 11, 27, 31, 39, 43, 62, 67, and 74, as well as CE families 1 and 5, were common across carbon sources and time points. DEGs related to pectin degradation were observed within the GH families 28, 78, and 105, CE families 8 and 12, and PL families 1 and 4. Exclusive up-regulated genes were observed on SH, namely for a gene encoding an exopolygalacturonase GH28 member (ATEG 07152), for an alpha-L-rhamnosidase GH78 member (ATEG 02922 and a Rhamnogalacturonate lyase B (PL4 family) (ATEG 10327). Two genes belonging to the CE families 8 and 12 were common across carbon sources and time points. Lytic polysaccharide monooxygenases (LPMOs) were also observed amongst the DEGs, with one AA3 gene and five AA9 genes up-regulated on both SB and SH. Exclusive up-regulated AA3-encoding (ATEG 09993), AA7 (ATEG 07698) and AA9-encoding genes (ATEG 10194) were observed on SB.

### Expression of Transcription Factors

Out of a total of 205 transcription factor-encoding genes expressed during growth on SB and SH, a total of 15 were differentially expressed on SB or SH in comparison with equivalent growth periods on glucose as carbon source (log2 fold change (FC) at least ≥2-fold and at a probability level of *p* ≤ 0.01) (Supplementary Table 3). On the basis of genome annotation and FUNC-based analysis of Pfam and InterPro entries, these comprised gene orthologs encoding Zn_2_Cys_6_ transcription factors, C6 transcription factors (including AmyR), CP2 transcription factors, a bZIP transcription factor (AtfA), and a zinc finger Cys_2_His_2_ transcription factor. Where log2 fold change (FC) was <2-fold and at a probability level of *p* ≤ 0.01, a total of 84 displayed modulation during at least one of the four treatments on the two lignocellulosic carbon sources and growth time points. These comprised transcription factors annotated as Zn_2_Cys_6_ (Btf3, Hfs, tamA, SAGA, jumonji family, GATA-type, FilB, acuM, TPA, RfeB, bZip), zinc finger Cys_2_His_2_, C6, APSES / Homeobox KN domain, MADS box, heat shock, PHD, CP2, Ran-specific GTPase-activating protein, TFIIH subunit Tfb4, cellobiose response regulator, HLH, bZIP, GATA, homeobox, HTH/APSES/RfeD, CCAAT-binding HapE, TFIIIB complex subunit Brf1, TFIIH basal transcription factor complex p47 subunit, and hypothetical or unnamed protein transcription factor genes.

### Expression of Transporters

Analysis of expression of transporter genes across the four treatments during growth on SB and SH, in comparison to glucose, revealed considerable modulation according to carbon source (Supplementary Table 4 and Supplementary Figure 3). From a total of 124 transporter genes with significant differential expression (log2 fold change (FC) at least ≥2-fold and at a probability level of *p* ≤ 0.01), 60 were annotated as orthologs belonging to the protein major facilitator superfamily (MFS), in which the majority of sugar transporters are classified. Within these, candidate sugar transporter genes were classified as MFS glucose transporters, MFS hexose transporters, MFS monosaccharide transporters, and MFS sugar transporters. A total of 16 potential sugar transporters were significantly up-regulated on both SB and SH during the 48 h incubation period, namely ATEG_07791, ATEG_03475, ATEG_07105, ATEG_04988, ATEG_01556, ATEG_07124, ATEG_08653, ATEG_07144, ATEG_03527, ATEG_02489, ATEG_07114, ATEG_04070, ATEG_07053, ATEG_04137, ATEG_03190, and ATEG_05008.

### RT-qPCR Validation of *in silico* RNA-Seq Transcriptome Data

Expression profiles for selected *A. terreus* CAZyme-encoding and other genes significantly up-regulated on SB and SH in comparison to growth on G were examined by RT-qPCR in order to validate expression data based on Illumina RNA-Seq. These genes comprised endo-1,4-beta-xylanases (ATEG_07461, ATEG_03410, and ATEG_00809), an endoglucanase (ATEG_07420), a polygalacturonase (ATEG_04991), a membrane protein (ATEG_02687), and hypothetical or predicted proteins (ATEG_07383, ATEG_04652). Analysis generally revealed similar expression tendencies for both RT-qPCR and RNA-Seq data for each of the genes across the investigated growth treatments. Supplementary Figure 4 shows log2 Fold Change representations of expression modulation on each lignocellulosic carbon source in comparison to glucose, at each investigated time point. Differences in log_2_ FC values may occur due to the different algorithms applied for estimation of fold change with each approach. The primer sequences employed for RT-qPCR analysis of expression of each of the target genes are provided in Supplementary Table 5.

## Discussion

The potential of biorefinery systems in the 21st Century is considerable, not only in terms of the contribution to global sustainable energy supply in the transition to greenhouse gas neutrality, but also in terms of other bio-products (e.g., chemicals, amino acids, polymers) in a circular economy format that favors socio-economic development (King et al., 2010; Jungmeier et al., 2013; Silva et al., 2017). Countries with substantial agricultural industries, such as Brazil, produce large quantities of lignocellulosic waste, making them strategically placed to contribute to biorefining, either through the export of biomass materials or through in-country development of biorefinery hubs within agricultural zones. Whilst Brazil is already a global example for bioethanol and bioplastic production, optimization of enzymatic conversion of lignocellulose, as in all countries devoted to such white biotechnology, remains an obstacle to economic viability and sustainability, accounting, for example, for almost 30% of production costs for cellulosic bioethanol (Johnson, 2016).

Given the ability of certain filamentous Ascomycete fungi to secrete cellulases and other hydrolytic enzymes into growth media, prospection for efficient producing species has been extensive. In this context, investigation has been considerable into furthering understanding of the molecular mechanisms involved in lignocellulose biodegradation by filamentous fungi. In the case of the genus *Aspergillus*, lignocellulose biodegradation potential has been investigated at the transcriptome level in species such as *A. fumigatus* (Miao et al., 2015; Damasio et al., 2017; de Gouvêa et al., 2018), *A. niger* (de Souza et al., 2011; Delmas et al., 2012; Pullan et al., 2014; van Munster et al., 2014; Brown et al., 2016), *A. nidulans* (de Assis et al., 2015), *A. sydowii* (Cong et al., 2017) and *A. tamarii* (Midorikawa et al., 2018). Typically isolated from soil and decomposing lignocellulosic plant biomass (Reddy and Singh, 2002; He et al., 2004; Chantasingh et al., 2006), ligninolytic enzyme activities have been investigated in *A. terreus*, across diverse biorefinery applications, from bioethanol (Sethi et al., 2017), to lovastatin (Jahromi et al., 2012) and organic acid production (Jiménez-Quero et al., 2017). Analysis of the transcriptome of this fungus has, however, focused only on secondary metabolism related to biogenesis of lovastatin (Palonen et al., 2017), with no investigation, prior to this study, of global gene expression during hydrolysis of lignocellulosic plant biomass.

This analysis of the secretome and transcriptome of the fungus *A. terreus* BLU24 following growth on two important abundant agricultural lignocellulosic biomass residues in Brazil provides important information on CAZyme-encoding genes, transcription factors and transporter genes that are activated during the saccharification of these complex carbon sources. The significant upregulation of glycoside hydrolases, carbohydrate esterases, polysaccharide lyases, and auxiliary activities involved in cellulose, hemicellulose and pectin degradation observed following growth on SB and SH highlight the potential in this strain for lignocellulosic biomass degradation. Given the observed presence of additional gene sequences in *A. terreus* BLU24 that did not map to the reference genome, including, for example, those with identities to genes encoding exo- and endo-1,4-beta-xylanases, beta-glucosidases, 1,4-beta-D-glucan cellobiohydrolases and endo-1,4-beta-glucanases, further characterization of this strain at the genome level is also warranted.

Previous bromatological analyses of different lignocellulosic biomass residues as carbon sourced for induction of hydrolytic enzymes in filamentous fungi reported SH to be composed of 44.4% cellulose, 16.7% hemicellulose and 5.2% lignin. Values were somewhat reduced in SB, with 34.6% cellulose, 16.2% hemicellulose and 5.1% lignin (Siqueira, 2010). As shown in Figure 1, SEM observations of the cell walls of SB and SH revealed rapid colonization of these lignocellulosic materials and consistent with the ability of the fungus to secrete appropriate hydrolytic enzymes for biodegradation.

Xylanases (endo-β-1,4-xylanase; EC 3.2.1.8) are responsible for the random hydrolysis of B-(1,4) glycosidic bonds in xylan in plant cell walls. Xylans can comprise up to 35% of the dry weight of annual plants, with these enzymes widely applicable in the saccharification of biomass. Despite the higher cellulose content in the evaluated substrates, xylanase activities on SB and SH were considerably higher than those for CMCases, pectinases and FPases, both initially during the first 48 h and for the duration of the 9-day evaluated time period on each carbon source. In the plant cell wall, the interaction of xylan with cellulose microfibrils increases recalcitrance to cellulose active enzymes. The earlier production of xylanases observed here likely contributes to xylan degradation and subsequent increased substrate accessibility for CMCases and FPases. In previous studies in *A. niger* and *A. oryzae*, a clear relationship was observed between the presence of D-xylose and increased expression of cellulose-encoding genes, regulated by the transcriptional factor XlnR (de Souza et al., 2011; Tani et al., 2014). Here, our results may indicate a similar mechanism occuring in *A. terreus*, where an initial release of D-xylose following activity of xylanases results in subsequent increased secretion of CMCases and FPases for continued cellulose hydrolysis.

Given the previous reports on lignocellulolytic enzyme production in *A. terreus*, secretion of enzymes covering different CAZyme families and subfamilies was expected on the two complex lignocellulosic carbon sources employed in this study. In total, 89 CAZyme-encoding genes were expressed on SB and 91 on SH. These numbers highlight the wide spectrum of hydrolytic enzymes likely secreted and necessary for the hydrolysis of these complex carbon sources.

Global analysis of gene expression on the two lignocellulosic carbon sourced through GO enrichment analysis revealed enrichment for terms associated with carbohydrate degradation. The greater number of terms related to lignocellulose hydrolysis observed on SH rather than on SB suggests a larger arsenal of enzymes necessary for breakdown of this carbon source.

Considerable variation in CAZyme-encoding gene content can be observed across annotated reference genomes for *Aspergillus* species, with species such as *A. aculeatus*, *A. brasiliensis*, *A. carbonarius*, *A. luchuensis*, *A. nidulans*, *A. sydowii*, *A. tubingensis*, *A. versicolor*, and *A. wentii* containing 117, 435, 376, 410, 478, 501, 239, 539, and 387 predicted CAZyme-encoding genes involved in carbohydrate hydrolysis, respectively (Nordberg et al., 2014). In the case of *A. terreus*, from a total of 580 CAZyme-encoding genes predicted in the NIH 2624 reference genome, 475 encode families involved in hydrolysis. These include GHs (281), CBMs (91), AAs (57), CEs (27) and PLs (15).

The numbers of up-regulated CAZyme-encoding DEGs on SB and SH according to family reflect, to some degree, in terms of proportions, the numbers of such genes in the fungal genome. For both SB and SH, GHs were the most common up-regulated CAZyme-encoding DEGs, followed by CEs and AAs. The majority of the expected CAZyme families for a member-species of the genus *Aspergillus* were represented in the differentially expressed genes on both carbon sources (Segato et al., 2014). Whilst many of the CAZyme-encoding DEGs were up-regulated on both carbon sources, clear differences between SB and SH were also apparent, with generally greater numbers of cellulose-encoding genes significantly up-regulated on SB and greater numbers related to pectinase activities on SH.

Many genes encoding different GH family proteins were up-regulated on both SB and SH, with the most abundant within the families GH3, 5, 10, 31, and 43. Similar up-regulation was previously observed in *A. tamarii*, where transcriptome analysis on SB in comparison to glucose revealed CAZyme-encoding DEGs within GH families 3, 5, 31, 43, and 92 (Midorikawa et al., 2018). de Gouvêa et al. (2018) also reported highly expressed genes in *A. fumigatus* on SB belonging to most of these GH families, when compared to growth on fructose. Genome-based prediction of fungal secretomes has also revealed predicted conserved enzyme profiles for families GH 3, 5, 10, and 43 across up to 14 species in taxonomic sections Aspergillus, Candidi, and Flavi for the genus *Aspergillus*, indicating likely conservation of such carbohydrate-active enzymes during speciation (Barrett et al., 2020).

Independently of the biomass employed for cultivation and growth period evaluated, a conserved core set of 41 CAZyme-encoding DEGs was also observed. In Figure 7, we present a model of the principal CAZyme families represented in *A. terreus* BLU24 in the transcriptome data during degradation of SB and SH. With regard to cellulose, cellobiohydrolases, endoglucanases, glucosidases, LPMOs, and cellobiose dehydrogenases are required for degradation. Within the up-regulated CAZyme-encoding DEGs, all such cellulases were differentially up-regulated on SB or SH at least at one of the two investigated time points. Of these, genes belonging to the GH families 7, 12, 15, and 71 were up-regulated during all four treatments, indicating potential conservation in function in lignocellulose hydrolysis in this fungus. Previously, gene models for cellulose-degrading CAZymes within the GH family 7 have been described for the *Aspergillus* species *A. clavatus*, *A. nidulans*, *A. niger*, *A. terreus*, *A. fumigatus*, *A. flavus* and *A. oryzae*, and for GH12 for *A. clavatus*, *A. niger*, *A. terreus*, *A. fumigatus*, *A. flavus*, and *A. oryzae* (Segato et al., 2014 and references therein). Genome-based prediction has also been made for the families GH 7 and 12 in 15 species in *Aspergillus* sections Aspergillus, Candidi, and Flavi (Barrett et al., 2020). Greatest numbers of CAZyme-encoding DEGs were observed for those involved in hemicellulose degradation, corroborating the high xylanase enzyme activities observed in this fungus on both SB and SH. Hemicellulose represents a more complex structure than cellulose, with the presence of different monomers (pentoses and hexoses) and ramifications. As a consequence, greater numbers of enzymes are required for hemicellulose hydrolysis, comprising xylanases, arabinofuranosidases, alpha-glucuronidases, xyloglucanases, arabinanases, mannosidases or mannanases, acetyl xylan esterases, feruloyl esterases, xylosidases. Of the up-regulated CAZyme-encoding DEGs, all such enzymes, with the exception of feruloyl esterases, were differentially up-regulated on either SB or SH at least during one of the time points. Genes belonging to the GH families 3, 5, 10, 11, 27, 31, 39, 43, 62, 67, and 74, and CE families 1 and 5 were up-regulated during all four treatments, again indicating potential conservation in lignocellulose hydrolysis. Of these, genome-predicted enzyme profiles have been described previously for xylan-degrading enzymes in GH families 3, 10, 11, 43, 62 and 67, and in CE families 1 and 5, again, in up to 15 species in *Aspergillus* sections Aspergillus, Candidi and Flavi (Barrett et al., 2020). Mannan polysaccharides are the second most abundant hemicelluloses after xylan, with their conversion of considerable interest for the biorefinery industry (Soni and Kango, 2013). With previous purification and characterization in *A. terreus* (Soni et al., 2016), here we observed four CAZyme-encoding DEGs associated with degradation of these polysaccharides. Whilst the pectin content in SB and SH is considerably lower than for hollocellulose (de Souza Moreira et al., 2013; Li et al., 2017), evidence was also observed for pectin degradation on both, with considerable exclusive up-regulation of genes on SH likely reflecting differences in pectin content between the different carbon sources. Up-regulation of genes related to pectinolytic activity have also been reported previously on SB for *A. niger* (PL4 family), *A. fumigatus* (PL4 family) and for *A. tamarii* (PL1, PL9, GH28 families) (Borin et al., 2017; de Gouvêa et al., 2018; Midorikawa et al., 2018). Genome-predicted enzyme profiles in *Aspergillus* sections Aspergillus, Candidi and Flavi indicate the presence of pectin-degrading enzymes in the GH families 28, 43, 53, 78, and 142, in the CE family 8, and in the PL families 1, 3, 4, and 26, again with considerable conservation in up to 15 species (Barrett et al., 2020). Here, up-regulated CAZyme-encoding DEGs were observed for members of GH families 28, 78, and 105, for PL families 1 and 4, and for CE families 8 and 12, with the latter two up-regulated across all four growth treatments. As pectin also acts as a barrier that limits access of cellulase to their substrates, the secretion of pectin active enzymes might guarantee more efficient substrate degradation by *A. terreus*.

Lytic polysaccharide mono-oxygenases, which are classified into CAZy auxiliary activities families AA9-AA11 and AA13-AA16, are copper-dependent enzymes that also perform important roles in lignocellulose degradation, reducing recalcitrance of less accessible regions of the plant cell wall through oxidative cleavage of glycosidic bonds, facilitating cellulose recognition by cellulases and boosting efficiency in hydrolysis of biomass (Monclaro and Filho, 2017). These enzymes can also depolymerize hemicellulosic polymers (Couturier et al., 2018), thus providing an overall enhancement to the polysaccharide conversion process, working in synergy with glycoside hydrolases. With AA9, AA11, AA13, AA14, and AA16 exclusive to fungal genomes, multiple genes encoding LPMOs appear to be common in fungal genomes, particularly in Ascomycetes and Basidiomycetes (Kracher et al., 2016). With previous reports of LPMOs in transcriptome and/or secretomes of *A. niger*, *A. fumigatus, and A. tamarii* following growth on sugarcane bagasse (Borin et al., 2015, 2017; de Gouvêa et al., 2018; Midorikawa et al., 2018), and recent characterization of seven AA9s in the secretome of *A. terreus* on soybean spent flakes (Pierce et al., 2017), investigation here also highlights their involvement in the degradation of the SB and SH, with one AA3 gene and five LPMO AA9 genes up-regulated on both SB and SH lignocellulosic carbon sources, as well as exclusive AA3, AA7 and AA9-encoding genes on SB. Such data highlights potential conserved functional roles in biomass degradation, as reported in other fungi (Isaken et al., 2014), as well as evidence for substrate specific activity, as documented recently for AA9 LPMOs (Jagadeeswaran et al., 2016; Hüttner et al., 2019). These advances in our knowledge of the LPMO enzyme repertoire in *A. terreus* are relevant for improving efficiency in lignocellulosic biomass degradation.

Transcription factors play major roles in the coordination of gene expression in the cell. To date, numerous transcription factors in different fungi have been reported to be involved in plant biomass degradation, modulating the expression of plant cell wall degrading enzymes (Wu et al., 2020). Activation of such transcription factors likely occurs following hyphal contact with low molecular weight mono-or disaccharide compound derivatives of complex polymers (Benocci et al., 2017). The majority that have been characterized to date belong to the zinc binuclear cluster family, with most positive expression regulators belonging to the Zn_2_Cys_6_ class (Todd et al., 2014). In *Aspergillus* spp. these include XlnR, a key regulator of (hemi)cellulose degradation, together with AraR, involved in L-Arabinose utilization, Clr-A and Clr-B (cellulose degradation), AmyR (starch degradation), MalR (maltose utilization), ClbR (cellobiose utilization), RhaR (pectin deconstruction / L-Rhamnose utilization), GaaR (pectin deconstruction/galacturonic acid utilization), InuR (inulin utilization), and GalX and GalR (D-galactose utilization). Other important transcription factor classes controlling gene expression in *Aspergillus* spp. related to biomass degradation comprise CreA (carbon catabolite repression), within the Cys2His2 class and the HAP complex (Cazy regulation), within the CBF class (Benocci et al., 2017 and references therein). Whilst mechanisms for regulation of expression of sugar transporter genes during sugar utilization in fungi is only partially understood at present, a number of the above transcription factors, such as XlnR, CreA, ClrA/ClrB, GalX and AraR, are also known to regulate sugar transporter genes in model *Aspergillus* species (e. g. Vankuyk et al., 2004; Andersen et al., 2008; de Souza Moreira et al., 2013). Our data revealed considerable numbers of transcription factor genes with significant differential expression on SB and/or SH at a probability level of *p* ≤ 0.01, with 15 at log2 fold change (FC) at least ≥2-fold and 84 at log2 fold change (FC) <2-fold. Those with significant differential expression and previously reported as involved in plant biomass utilization in ascomycete fungi included AmyR (starch degradation), Ctf1B (cutinase induction / cellulase, xylanase production) and AreB (nitrogen metabolite repression). With many classified, based on the available genome annotation data, as members of the important Zn_2_Cys_6_ class, further work is warranted into accurate prediction, annotation and function validation in *A. terreus* BLU24.

The majority of sugar transporters belong to the sugar porter family within the major facilitator superfamily. Sugar transporters are essential proteins in lignocellulolytic fungi that enable the uptake of mono- and short oligosaccharide products of extracellular enzymatic digestion of lignocellulose into the fungal cell for growth and metabolism. Although likely to be abundant in fungal genomes, especially in those species adapted to lignocellulose decomposition, their comprehensive investigation has been restricted mostly to model organisms such as *Saccharomyces cerevisiae* (Wieczorke et al., 1999). Within the genus *Aspergillus*, sugar transporter genes have recently been characterized in species such as *A. niger*, with 86 putative genes identified *in silico*, based on proteome and transcriptome analysis (Peng et al., 2018), as well as *A. nidulans*, with 357 proteins in the major facilitator superfamily predicted in the annotated genome, albeit without validation of roles in sugar transport for the majority (de Gouvêa et al., 2018). Lignocellulose contains both hexose (glucose) and pentose (mostly xylose) sugars which can be released by the enzymatic action of ligninolytic fungi. Biorefinery of lignocellulose is limited in part by the inability of many fermenting microorganisms to metabolize sugars other than glucose (Young et al., 2010). The characterization of additional sugar transporters, therefore, such as those for xylose and cellobiose transport, which are modulated on lignocellulose, may therefore enable internalization of these additional sugars to overcome such limitations. Transformation of *S. cerevisiae* with cellobiose and xylose transporters from *A. nidulans*, for example, has highlighted the potential in the use of such genes from *Aspergillus* sp. for genetic engineering of yeasts for complete fermentation of all sugars in lignocellulose for conversion to bioethanol (dos Reis et al., 2016). Prior to this study, there have been no reports on sugar transporter modulation in *A. terreus* during lignocellulose breakdown. Here we identified 16 potential sugar transporters within the major facilitator superfamily that were up-regulated on both SB and SH during the 48 h incubation period, indicating the likely release of sufficient fermentable sugars such as glucose, xylose or cellobiose to result in induction of their expression during hydrolysis of these complex carbon sources. Such genes offer potential in genetic engineering to optimize non-glucose pentose sugar transport in fermenting organisms.

Lignocellulosic plant biomass saccharification in biorefinery applications using thermostable enzymes offers advantages in terms of efficiency, with increased catalytic activity in hydrolysis, solubilization of lignocelluloses, lower contamination, lower cooling requirements and increased flexibility in fermentation process design (Sonnleitner and Fiechter, 1983; Sharma et al., 2014). Although numerous fungal genera contain species with the ability to secrete enzymes stable at temperatures above 60 °C, such as *Sporotrichum*, *Chaetomium*, and *Humicola*, amongst others, growth rates of these mesophilic fungi on lignocellulosic substrates are typically low, with cellulases produced in only low titers. Investigation of thermophilic fungi, by contrast, has reported faster growth rates and more rapid cellulose degradation, even at lower pH values (Turner et al., 2007). Cellulase activity in *A. terreus* has been shown to be acidothermophilic (e.g., Gao et al., 2008), with optimal activities also reported between 50 and 60°C, at pH 4.0–6.0 (Sharma et al., 2014). Similarly, high xylanolytic activity has been observed over a wide pH range from 3.0 to 10.0 (Chantasingh et al., 2006), appropriate for employment in bio-bleaching for paper pulp (Filho, 1998). With LPMOs also reported to be stable under low pH conditions (Agrawal et al., 2020), as well as mannanases (Soni et al., 2016), such thermostable properties of enzymes from *A. terreus* are therefore advantageous for industrial biorefinery applications of lignocellulose saccharification.

This first investigation of the transcriptome of *A. terreus* following growth on two abundant lignocellulosic biomasses from abundant commodity crops provides important functional genomics information on the network of CAZyme encoding genes, transcription factors and sugar transporter genes involved in the enzyme hydrolysis of these complex carbon sources. This advance in our understanding of the biology of the fungus is applicable in the genetic improvement of both promising fungi for lignocellulosic carbon hydrolysis and yeasts involved in hexose and pentose sugar fermentation steps in biorefineries.

## Data Availability Statement

The datasets presented in this study can be found in online repositories. The names of the repository/repositories and accession number(s) can be found in the article/Supplementary Material.

## Author Contributions

RM, EN, and EF planned the experiments. GM and CC performed the bioassays, enzyme analyses, RNA and cDNA preparation, and sequence data analysis. RT, MC, GA, OS-J, and PG participated in sequence data analyses and in editing of the manuscript. RM conceived the study, participated in bioassays, RNA preparation for cDNA library construction, sequence data analysis, and drafted the manuscript. All authors contributed to read and approved the final version of the manuscript.

## Conflict of Interest

The authors declare that the research was conducted in the absence of any commercial or financial relationships that could be construed as a potential conflict of interest.

## Footnotes

1 http://www.cazy.org

2 http://www.broadinstitute.org

3 http://www.novocraft.com/documentation/novoalign-2/novoalign-user-guide/rnaseq-analysis-mrna-and-the-spliceosome/

4 http://revigo.irb.hr/

5 https://www.idtdna.com/calc/analyzer

## Supplementary Material

Supplementary Figure 1 Selected gene ontology (GO) enrichment analysis data for differentially expressed genes in *Aspergillus terreus* BLU24 after 36 and 48 h growth on sugarcane bagasse (SB) and soybean hulls (SH) as sole carbon source compared with glucose-supplemented treatments. Significant differentially expressed genes were considered if a log2 fold change (FC) was at least ≥2-fold and at a probability level of *p* ≤ 0.01. Enrichment values were compared with those occurring in the *A. terreus* NIH 2624 reference genome sequence.

Supplementary Figure 2 Heatmap depiction of hierarchically clustered groups of *Aspergillus terreus* BLU24 CAZY genes according to gene expression modulation following growth on different carbon sources. Gene expression modulation was compared between the growth treatments SB36 and G36, SB48 and G48, SH36 and G36, and SH48 and G48. Statistically significant differentially expressed genes were considered if a log2 fold change (FC) was at least ≥2-fold and at a probability level of *p* ≤ 0.01. All FC values below −6 or above 6 were considered as minimum or maximum values, respectively.

Supplementary Figure 3 Heatmap depiction of hierarchically clustered groups of *Aspergillus terreus* BLU24 transporter genes according to gene expression modulation following growth on different carbon sources. Gene expression modulation was compared between the growth treatments SB36 and G36, SB48 and G48, SH36 and G36, and SH48 and G48. Statistically significant differentially expressed genes were considered if a log2 fold change (FC) was at least ≥2-fold and at a probability level of *p* ≤ 0.01. All FC values below −6 or above 6 were considered as minimum or maximum values, respectively.

Supplementary Figure 4 RT-qPCR validation of differential gene expression profiles based on RNA-Seq for selected genes in *Aspergillus terreus* BLU24. Standard error values (black bars) were calculated based on data generated from analysis of three biological replicates per treatment and three technical amplification replicates.

Supplementary Table 1 Illumina RNA-Seq data sequence statistics for each *Aspergillus terreus* BLU24 cDNA library.

Supplementary Table 2 Global gene expression data for each *Aspergillus terreus* BLU24 cDNA library following growth on SB and SH in comparison to equivalent growth on glucose as carbon source, together with annotation data for unmapped sequence reads aligned to the NCBI nr database.

Supplementary Table 3 Transcription factor genes in *Aspergillus terreus* BLU24 differentially expressed on SB and SH in comparison to equivalent growth treatments with glucose as carbon source.

Supplementary Table 4 Transporter genes in *Aspergillus terreus* BLU24 differentially expressed on SB and SH in comparison to equivalent growth treatments with glucose as carbon source.

Supplementary Table 5 Primer sequences for RT-qPCR validation of differential gene expression of selected target genes.
