# Supplementary figures and images for: Transcriptome Profiling-Based Analysis of Carbohydrate-Active Enzymes in Aspergillus terreus Involved in Plant Biomass Degradation
Source: Front Bioeng Biotechnol. 2020 Oct 6;8:564527. doi: 10.3389/fbioe.2020.564527 (PMC7573219; doi:10.3389/fbioe.2020.564527)

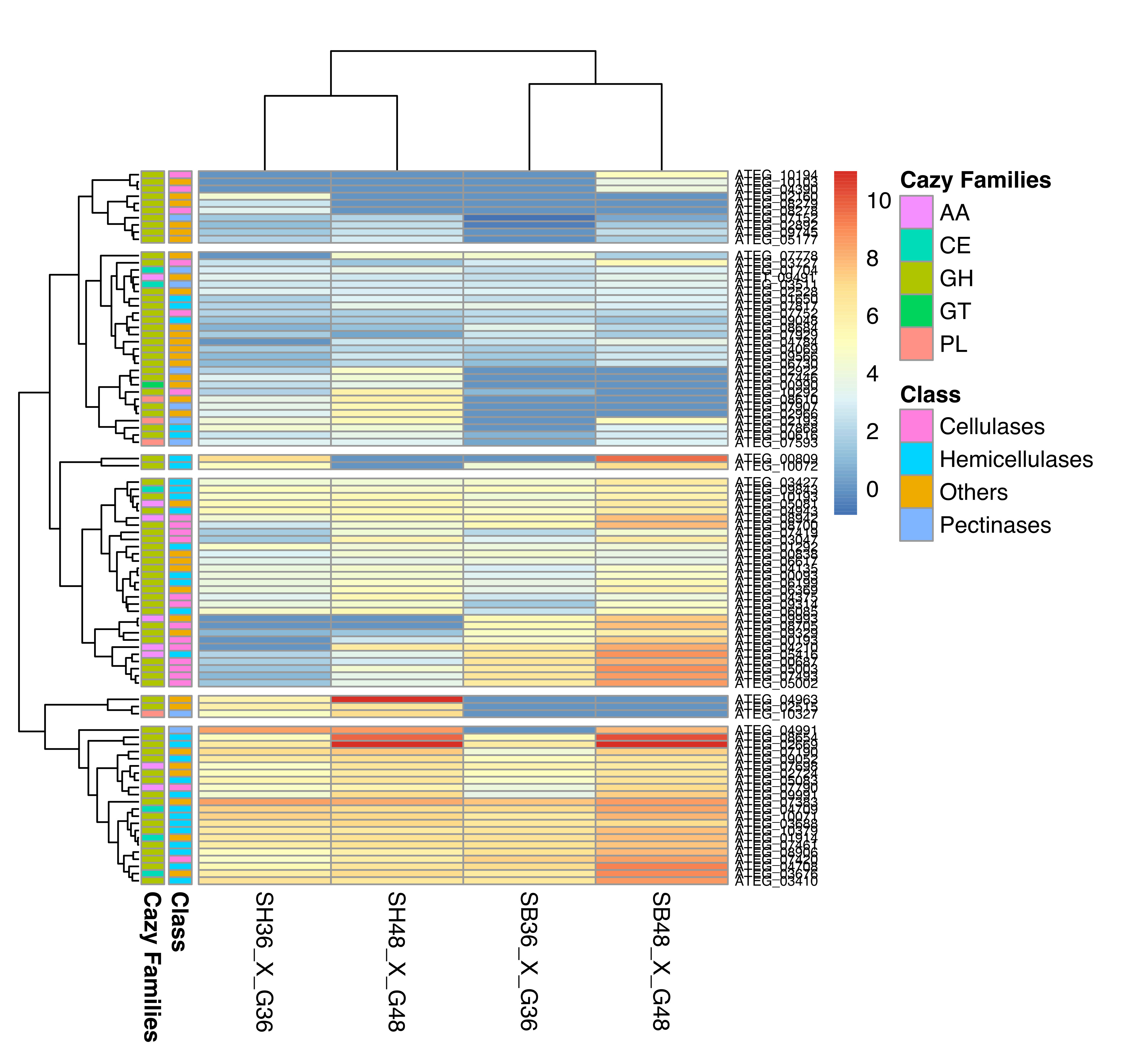

Supplement: Supplementary Figure 2 — Heatmap depiction of hierarchically clustered groups of Aspergillus terreus BLU24 CAZY genes according to gene expression modulation following growth on different carbon sources. Gene expression modulation was compared between the growth treatments SB36 and G36, SB48 and G48, SH36 and G36, and SH48 and G48. Statistically significant differentially expressed genes were considered if a log2 fold change (FC) was at least ≥2-fold and at a probability level of p ≤ 0.01. All FC values below −6 or above 6 were considered as minimum or maximum values, respectively. [file Image_2.JPEG]

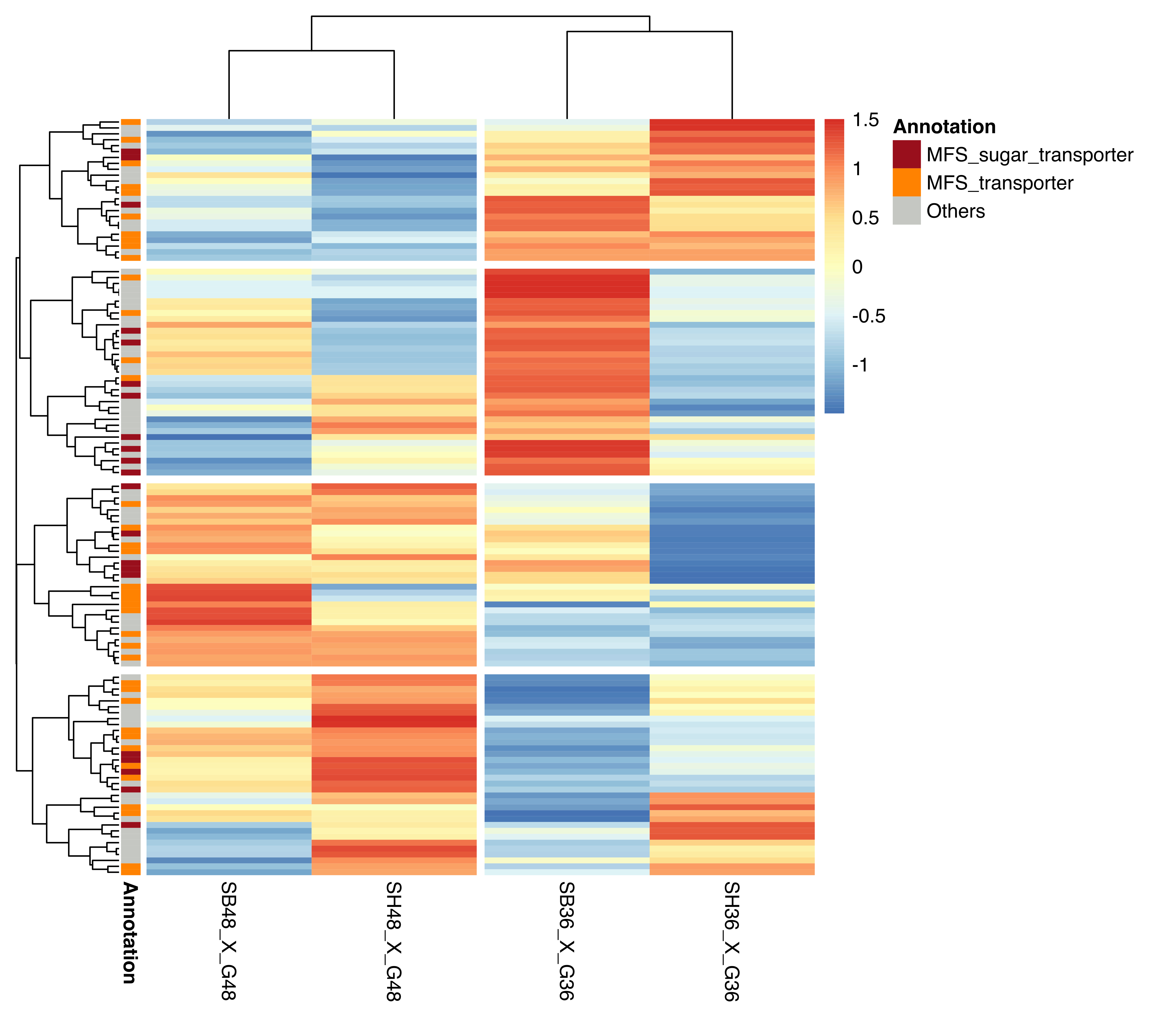

Supplement: Supplementary Figure 3 — Heatmap depiction of hierarchically clustered groups of Aspergillus terreus BLU24 transporter genes according to gene expression modulation following growth on different carbon sources. Gene expression modulation was compared between the growth treatments SB36 and G36, SB48 and G48, SH36 and G36, and SH48 and G48. Statistically significant differentially expressed genes were considered if a log2 fold change (FC) was at least ≥2-fold and at a probability level of p ≤ 0.01. All FC values below −6 or above 6 were considered as minimum or maximum values, respectively. [file Image_3.JPEG]

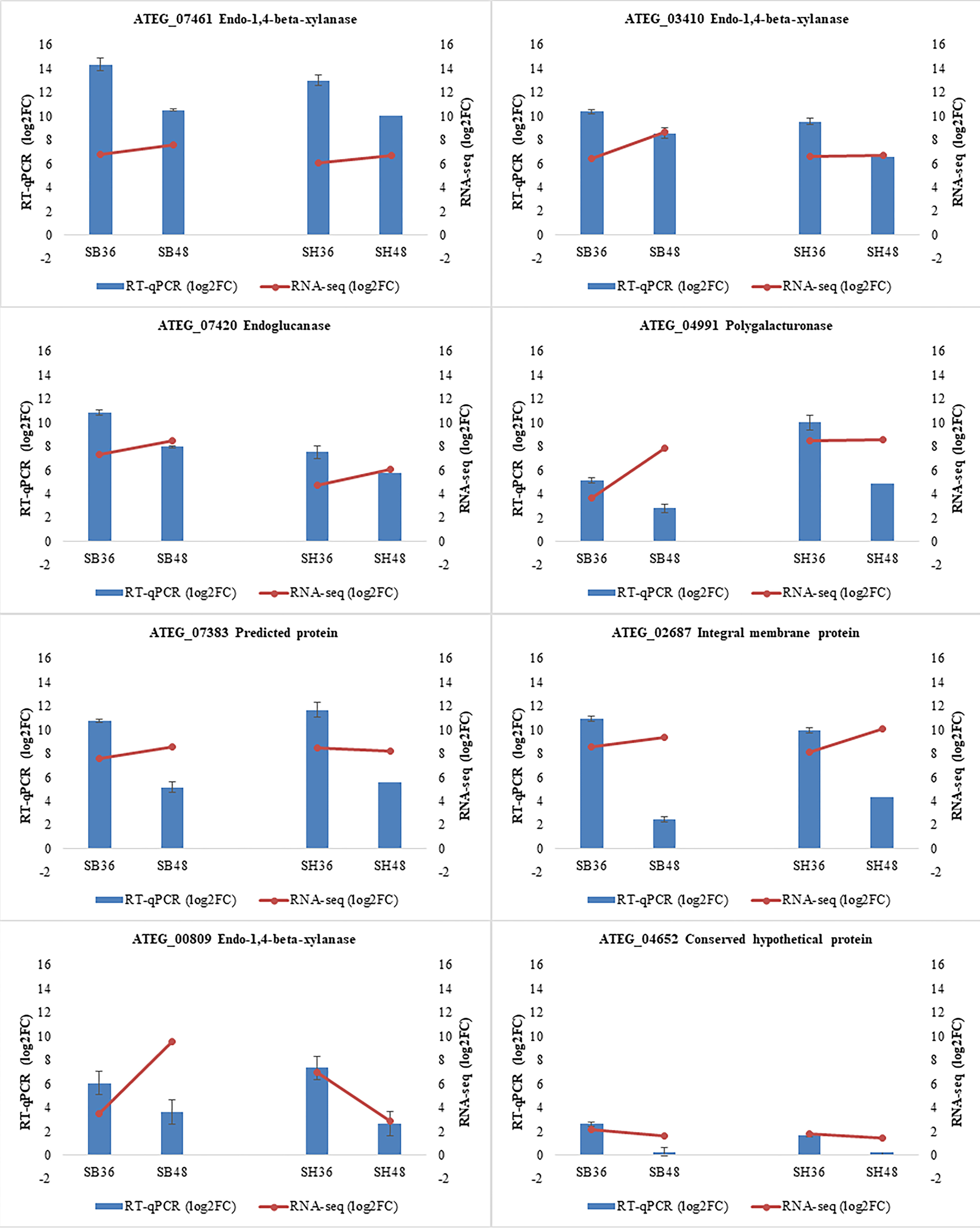

Supplement: Supplementary Figure 4 — RT-qPCR validation of differential gene expression profiles based on RNA-Seq for selected genes in Aspergillus terreus BLU24. Standard error values (black bars) were calculated based on data generated from analysis of three biological replicates per treatment and three technical amplification replicates. [file Image_4.JPEG]
