# Supplementary material for: Transcriptome Profiling-Based Analysis of Carbohydrate-Active Enzymes in Aspergillus terreus Involved in Plant Biomass Degradation
Source: Front Bioeng Biotechnol. 2020 Oct 6;8:564527. doi: 10.3389/fbioe.2020.564527 (PMC7573219; doi:10.3389/fbioe.2020.564527)
Supplement: Supplementary Table 1 — Illumina RNA-Seq data sequence statistics for each Aspergillus terreus BLU24 cDNA library. [file Table_1.DOCX]

**Supplementary Table 1:** Illumina Hiseq 2500 sequence data statistics for each *Aspergillus terreus* BLU24 cDNA library

| **Sample Code** | **Illumina Flowcell lane** | **Yield (Gbases) per library** | **Sequence reads (Million)** | **Percentage of reads with quality Fastq QC>30** | **Mean quality Fastq QC>30 score** |
| --- | --- | --- | --- | --- | --- |
| SB36rep1_1 | 1 | 2.97 | 29.67 | 81.03 | 32.41 |
| SB36rep2_1 | 1 | 3.06 | 30.56 | 80.97 | 32.37 |
| SH36rep1_1 | 1 | 2.77 | 27.66 | 80.92 | 32.37 |
| SH36rep2_1 | 1 | 2.86 | 28.63 | 80.64 | 32.29 |
| SB48rep1_1 | 1 | 2.41 | 24.08 | 80.86 | 32.36 |
| SB48rep2_1 | 1 | 2.41 | 24.14 | 81.54 | 32.56 |
| SH48rep1_1 | 1 | 2.49 | 24.85 | 80.82 | 32.33 |
| SH48rep2_1 | 1 | 3.16 | 31.56 | 81 | 32.39 |
| G36rep1_1 | 1 | 2.58 | 25.78 | 79.9 | 32.06 |
| G36rep2_1 | 1 | 2.29 | 22.86 | 80.11 | 32.14 |
| G48rep1_1 | 1 | 2.67 | 26.72 | 80.38 | 32.22 |
| G48rep2_1 | 1 | 2.96 | 29.55 | 81.2 | 32.45 |
| SB36rep1_2 | 2 | 2.91 | 29.11 | 82.26 | 32.76 |
| SB36rep2_2 | 2 | 3.08 | 30.75 | 82.24 | 32.74 |
| SH36rep1_2 | 2 | 2.8 | 27.98 | 82.17 | 32.72 |
| SH36rep2_2 | 2 | 2.88 | 28.84 | 81.91 | 32.65 |
| SB48rep1_2 | 2 | 2.4 | 23.98 | 82.15 | 32.73 |
| SB48rep2_2 | 2 | 2.41 | 24.09 | 82.81 | 32.92 |
| SH48rep1_2 | 2 | 2.51 | 25.1 | 82.08 | 32.69 |
| SH48rep2_2 | 2 | 3.15 | 31.47 | 82.29 | 32.76 |
| G36rep1_2 | 2 | 2.55 | 25.48 | 81.21 | 32.44 |
| G36rep2_2 | 2 | 2.33 | 23.29 | 81.47 | 32.52 |
| G48rep1_2 | 2 | 2.67 | 26.66 | 81.65 | 32.58 |
| G48rep2_2 | 2 | 3 | 29.97 | 82.45 | 32.81 |
