# Supplementary material for: Transcriptome Profiling-Based Analysis of Carbohydrate-Active Enzymes in Aspergillus terreus Involved in Plant Biomass Degradation
Source: Front Bioeng Biotechnol. 2020 Oct 6;8:564527. doi: 10.3389/fbioe.2020.564527 (PMC7573219; doi:10.3389/fbioe.2020.564527)
Supplement: Supplementary Table 5 — Primer sequences for RT-qPCR validation of differential gene expression of selected target genes. [file Table_5.DOCX]

**Supplementary Table 5.** Selected target genes and specific primer sequences for RT-qPCR validation of differential gene expression

| **Target Aspergillus / Genbank Gene ID** | **Predicted gene function** | **Forward Primer (5’-3’)** | **Reverse Primer (5’-3’)** |
| --- | --- | --- | --- |
| ATEG_07461 | Endo-1,4-beta-xylanase | TCTGCATGGCCTAG | GCATGTTGTGGCTGCAGTAG |
| ATEG_03410 | Endo-1,4-beta-xylanase | CTCAGCAACACGAACGACTT | GCGAGGTTGGCAATGGTAT |
| ATEG_07420 | Endoglucanase | AGCAGTTCTGGAACTATCTCG | AAACCCGACACAGTCATCG |
| ATEG_04991 | Polygalacturonase | CCCATCAAGGATGTCACTCTG | ACATTCTTCCAGGTCCAGTTG |
| ATEG_07383 | Predicted protein | ACATCAACCTTCGCCCTAAC | CTCCCTGTTCAAAGTCTCTTCC |
| ATEG_02687 | Integral membrane protein | CAGGCGGACAATATCGAAGAT | CCCAATATCATCACCGACTTGA |
| ATEG_00809 | Endo-1,4-beta-xylanase | TTCACCAGGTTCACCATACC | CGTCATCGGAGAAGACTACATC |
| ATEG_04652 | Conserved hypothetical protein | GGACTGTCGCTGAATGATGT | GCACGTAAGGTGACGTGATAA |
|  | *rDNA 18S | AACGGGTAACGGGGAATTAG | TGTCGGGATTGGGTAATTTG |
|  | *Actin | ACGAGAACGAGTTGAAAACG | ATAATTTGGGCCGCTGTATC |

*Gene expression data was normalized against stable reference genes for *Aspergillus terreus*
